# Habitat geometry in artificial microstructure affects bacterial and fungal growth, interactions, and substrate degradation

**DOI:** 10.1038/s42003-021-02736-4

**Published:** 2021-10-26

**Authors:** Carlos Arellano-Caicedo, Pelle Ohlsson, Martin Bengtsson, Jason P. Beech, Edith C. Hammer

**Affiliations:** 1 Department of Biology, Lund University, Lund, Sweden; 2 Department of Biomedical Engineering, Lund University, Lund, Sweden; 3 Division of Solid State Physics, Lund University, Lund, Sweden

**Keywords:** Bacteria, Fungal ecology, Soil microbiology, Microbial ecology, Microbial ecology

## Abstract

Microhabitat conditions determine the magnitude and speed of microbial processes but have been challenging to investigate. In this study we used microfluidic devices to determine the effect of the spatial distortion of a pore space on fungal and bacterial growth, interactions, and substrate degradation. The devices contained channels differing in bending angles and order. Sharper angles reduced fungal and bacterial biomass, especially when angles were repeated in the same direction. Substrate degradation was only decreased by sharper angles when fungi and bacteria were grown together. Investigation at the cellular scale suggests that this was caused by fungal habitat modification, since hyphae branched in sharp and repeated turns, blocking the dispersal of bacteria and the substrate. Our results demonstrate how the geometry of microstructures can influence microbial activity. This can be transferable to soil pore spaces, where spatial occlusion and microbial feedback on microstructures is thought to explain organic matter stabilization.

## Abstract (web summary)

Arellano Caicedo et al. use microfluidic devices to determine how spatial distortion of pore spaces in soil organic matter affects bacterial and fungal growth and substrate degradation. Their newly conceptualized chip design allows the quantification of bacterial and fungal biomass, and ecosystem function organic matter cycling with help of fluorescent markers in different microstructures.

## Introduction

Global mean temperatures are expected to keep increasing through the 21st century if current emissions of greenhouse gases are not lowered^1^. Soils contain more organic carbon than the atmosphere and the global vegetation together^2^, hence it has been urged by the Kyoto Protocol on climate change in 1992 for deeper understanding on the stabilization of carbon in soils^3^. The spatial micro heterogeneity in soils is thought to explain many of its unique properties, such as organic carbon storage^4–6^, where the heterogeneous distribution of pores, nutrients, air, and gas enhances microbial diversity and functions.

Microbes determine the biogeochemical cycles across all ecosystems^7^ where changes in microbial metabolism can lead to an acceleration or a delay of such cycles. It has been hypothesized that spatial restriction plays an important role in preventing microbial access to soil organic matter (SOM)^8^ which could explain the persistence of large amounts of SOM despite being largely composed of simple and highly nutritious molecules^9^. Since SOM constitutes a carbon stock that is larger than the combined stocks of the atmosphere and global vegetation^10^, changes in its carbon cycling rates will have consequently large effects on atmospheric CO_2_ levels. However, our understanding of how physical microstructure of soils affects microbes and their ecosystem functions, such as SOM turn over, is limited^11^.

Morphology and topology of the soil pore space have been characterized using direct methods of visualization, such as electron microscopy and X-ray tomography^12–15^. X-ray tomography studies have revealed correlations between pore size and SOM losses, feedback of SOM decay on the porous space, and an influence of pore heterogeneity, and their connection to the atmosphere as determinants of SOM fate^16–20^. Studies using these techniques have, however, limitations such as the lack of a controlled and manipulatable environment, the lack of real-time measurement of processes in the inner space, disturbance of natural conditions during sample preparation, subjectivity when thresholding greyscale images, and a current resolution limit of a few tens of micrometers which does not allow the study of smaller micropores^21^.

A complementary approach that solves these issues is to study soil pore space using model systems. Such model systems include the use of a controlled assembly of different soil materials^22^, transparent materials^23^, or 3D-printed soil structure proxies^23^. A particularly promising approach is micro-engineered or microfluidic devices^24^.

Microfluidics is defined as the manipulation of fluids within structures at the micrometer scale^25^. The use of microfluidics opens up the possibility of studying a wide range of microbial phenomena at the micrometer scale in a higher level of detail than with other methods. Some of the microbial processes studied with microfluidics include chemotaxis^26–29^, bacterial motility^27^, the effect of EPS in soil drying processes^30^, transport of nanoparticles by unicellular eukaryotes^31^, and fungal–bacterial interactions^32^. These systems can also be powerful tools to investigate the effect of soil physical characteristics on soil microbial communities^24^. For instance, we have shown earlier how different geometrical features in microfluidic devices, such as channel diameter and turning angles, can affect the distance various litter decomposer fungi can colonize within microfluidic chips^33,34^. Here, we are able to quantify biomass production of both fungi and bacteria via fluorescent markers, within a wider variety of turning angles. Further, we even quantify nutrient degradation activity via a fluorescent substrate distributed within the different microstructures. However, quantification of the impact of a wider variety of turning angles on fungal biomass, or on other organisms such as bacteria, their interactions, and on the nutrients they consume, has not been done before.

In the present study we have developed a microfluidic approach to explore the effect of a simulated pore space, consisting of differently angled channels, on fungal and bacterial biomass distribution and organic matter degradation. We chose to use the geometrical structures of long channels differing in their deviation from a straight, undisturbed passage that could represent major patterns found in the soil pore space. The studied channels differed in their bending angle, and the turning direction of the angles, which resulted in different tortuosities (here quantified as the ratio of the channel length to the straight distance between the beginning and the end of it). Angles were selected to represent the three main types of angles that can exist within a range of 180 degrees (acute, right, and obtuse). To track substrate degradation, we used a fluorogenic peptide that becomes fluorescent after enzymatic cleavage. The selected substrate is degraded only by the bacterial and not the fungal strain used, which allows us to estimate how bacterial substrate degradation is affected through the experiment. Our hypotheses were: (1) Bacterial and fungal biomass will be most strongly reduced in channels with sharp turning angles and repeated turn order. This, we thought, would occur because obstacles reduce fungal growth, according to models based on tomography images^35^ as well as because sharp angles require sharper turns than the most common natural turns of free-swimming bacteria^36^. (2) When growing together, we expected an enhancing effect of the angle and turn order on bacteria and fungi, respectively, because the presence of the other organism’s biomass contributes to the solid physical structure, thus increasing the complexity of the spatial structures. Finally, we hypothesized that (3) bacterial substrate degradation would follow bacterial biomass patterns and thus be higher in channels with smooth angles and alternated turns.

Data shows that both bacterial and fungal biomass are reduced in channels with sharper turning angles. The effect on fungi, however, is stronger than in bacteria for its biomass is dramatically reduced in sharp-angle channels. The amount of substrate consumed was reduced in sharp-angle channels only when bacteria and fungi were cultivated together. These results show the negative effect that sharp turning angles have on the growth of bacteria and fungi and on the nutrient degradation.

## Results

**Fig. 1:**
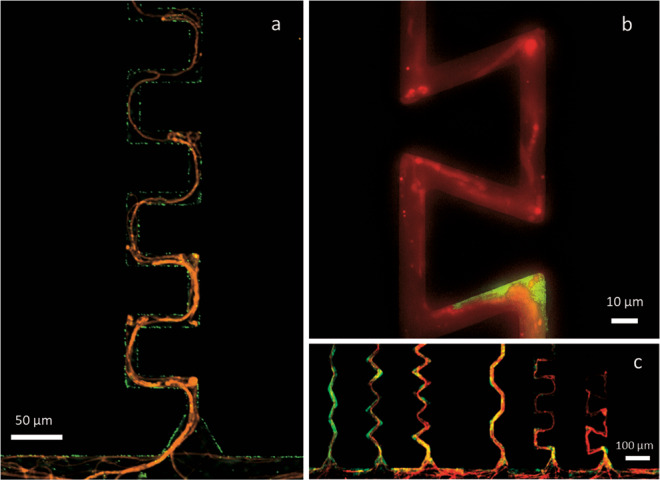
Epifluorescence images of the bacterial strain *P. putida* mt-2 (green) grown together with the fungal strain *C. cinerea* AmutBmut PMA412 (red) in amended M9 liquid medium inside the PDMS chip on day 2 after inoculation. **a** 90°-angled channel with repeated turn order. **b** 109°-angled channel with repeated turn order where fungal hyphae block the channel and do not allow bacteria to advance further. **c** All studied channel types (from left to right: 45°, 90°, and 109°, with alternated turning order, and 45°, 90°, and 109°, with repeated turning order) at 4x magnification.

We inoculated the chips with either a fungal (*Coprinopsis cinerea)*, a bacterial *(Pseudomonas putida)*, or a fungi+bacteria inoculum. The chips contained six types of channels that varied in two parameters: bending angle (45°, 90°, 109°) and bending order (alternated, repeated). Bacterial and fungal growth, together with substrate consumption (L-Alanine 7-amido-4-methylcoumarin trifluoroacetate salt), were followed over time using fluorescence microscopy. During the 14 days the experiment lasted, the organisms successfully colonized all chip channels of all tested turn angles and turn orders (Fig. 1a–c, Supplementary Fig. 1a–d) and caused measurable substrate consumption along the channels (Supplementary Fig. 2a–c, Supplementary Data 1).

### Bacterial biomass

**Fig. 2:**
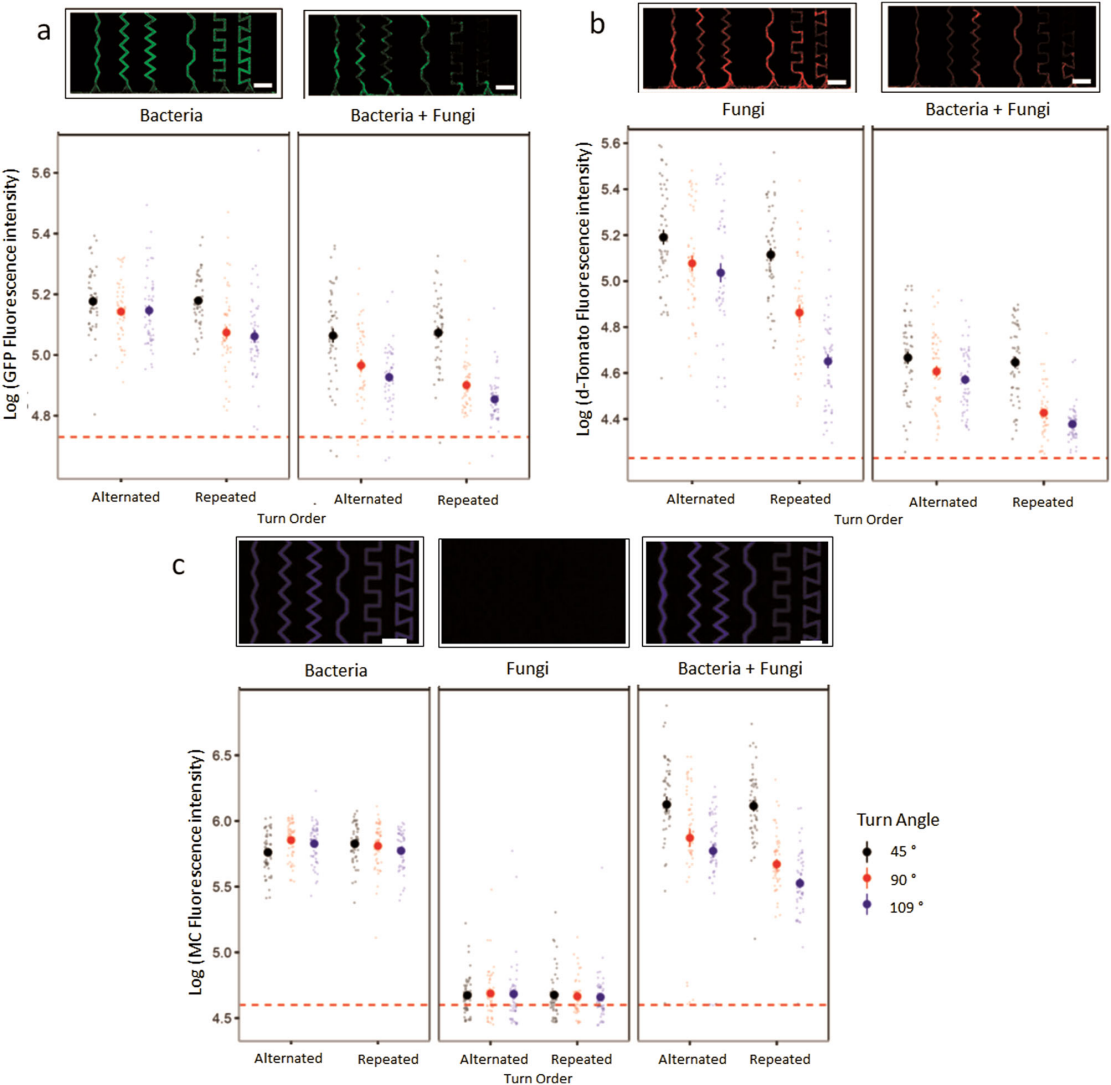
Bacterial and fungal biomass and their substrate consumption in differently angled channels. **a** Upper pannels show examples of the initial part of the microfluidic channels colonized by *P*.*putida* expressing GFP constitutively inside the microfluidic chip, on day 2 after inoculation, growing without (left) and with (right) the competitor *C. cinerea* (scale bar = 100 µm). Bottom panels show the three-way analysis of the response of bacterial biomass to the different channel types at day 2, in the absence (left) and in the presence (right) of the fungal competitor *C. cinerea*, as quantified via bacterial GFP fluorescence expression. **b** Upper pannels show examples of the initial part of the microfluidic channels colonized by *C.cinerea* expressing d-Tomato constitutively, on day 6 after inoculation, growing in the absence (left) and the presence (right) of the competitor *P. putida*. Below shows the three-way analysis plot of the d-Tomato fluorescence intensity of *C. cinerea* inside the channels on day 6, without (left) and with (right) the competitor *P. putida*. **c** Upper panels show the substrate consumption represented by fluorescence of released 4-methylcoumarin in the initial part of the microfluidic channels, at day 6 after inoculation with the bacterial strain *P. Putida* (“bacteria”), the fungal strain *C. cinerea* (”fungi”), and the combined experiment containing *P. putida* and *C. cinerea* (“bacteria + fungi”; Scale bars = 100 µm). The circles represent the mean log-transformed fluorescence of the respective fluorophore for each treatment and the error bars represent the ± SE based on ANOVA for all the channel types (*n* = 50). The dots correspond to each fluorescence measurement inside each type of channel. Color coding of dots and circles represent the angle of the channels, being black for 45°, red for 90°, and blue for 109°. The dashed red line represents the mean background fluorescence for each type of fluorescence channel.

While all channels were colonized over their whole length, there were significant differences in the amount of bacterial biomass depending on the angle and order of their turns (Fig. 2a). Angles reduced bacterial biomass as they became sharper. However, their effect was stronger when the bending order was repeated and when the fungus was present (Fig. 2a, Supplementary Table 1, Supplementary Table 2, Supplementary Table 3, Supplementary Fig. 3a). Repeated bending order lowered bacterial biomass, independently of whether the fungus was present or not. Alternated and repeated turn order channels differed significantly from each other in their impact on bacterial biomass only at angles of 90 and 109 degrees (Supplementary Fig. 3b) while they did not differ at angles of 45 degrees.

When the fungus was present, the effect of channel turning angles on bacterial biomass was increased, producing significantly lower bacterial biomass as angles became sharper. Such reduction occurred regardless of their angle turn order. Figure 1b shows an example of fungal hyphae blocking the access of bacteria to the deeper part of the channel.

Bacterial biomass was also negatively correlated with channel tortuosity in the presence and the absence of the fungal strain (Supplementary Fig. 4a, Supplementary Table 4).

### Fungal biomass

**Fig. 3:**
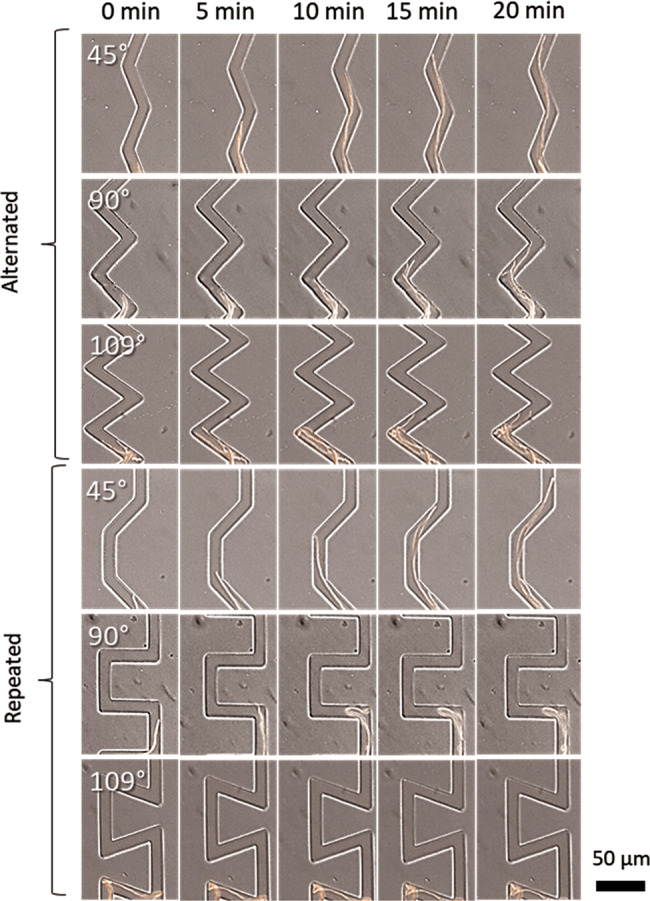
Time lapse images of *C. cinerea* passing through the different types of channels in the absence of a competitor at day 1. Pictures were taken with a 40X objective at a time interval of 5 min in brightfield with DIC overlayed with the red fluorescence channel.

Angles reduced fungal biomass in general as they became sharper, but their impact was significantly higher when their turn order was repeated (Fig. 2b, Fig. 3, Supplementary Table 5, Supplementary Table 6, Supplementary Table 7). The presence of bacteria caused a reduction of the overall fungal biomass independently of the structures, meaning that the fungal biomass was equally lower in every channel type when growing with the competitor compared to the corresponding channel when growing alone. This suggests that the effect bacteria had on fungal growth was not due to an interaction with structures that could lead to a habitat modification (Supplementary Fig. 5). The fungal biomass was distributed heterogeneously along the different types of channels, where typically most of the hyphae concentrated in the first parts of the channels, while fewer, if any, hyphae progressed towards the deep interior of the channels. The dispersal distance into the channels was especially decreased in sharp angle and repeated turn order channels (Fig. 3). Supplementary Movies 1–6 and Fig. 3 show the effect of every type of angle and turn order on the fungal growth.

Fungal biomass was negatively correlated with channel tortuosity (Supplementary Fig. 4b, Supplementary Table 8) and had a steeper slope when regressed against tortuosity in the absence of bacteria.

### Substrate consumption

Bacteria caused degradation of the AMC substrate detectable within 24 h, while fungi did not cause significant degradation during the experiment. The effect that the structures had on bacterial substrate degradation depended on the interactions with the fungi present in the chip (Fig. 2c). Bacteria alone had a generally higher substrate consumption than fungi alone in all types of channels and was not affected by the turn angles or the turn order of the channels (Supplementary Tables 9–11).

Only when bacteria were cultivated with fungi the AMC degradation was significantly affected by both the turn angle and turn order (Supplementary Fig. 6), generally showing decreasing substrate degradation as turn angles became sharper, and when turn order was repeated.

**Fig. 4:**
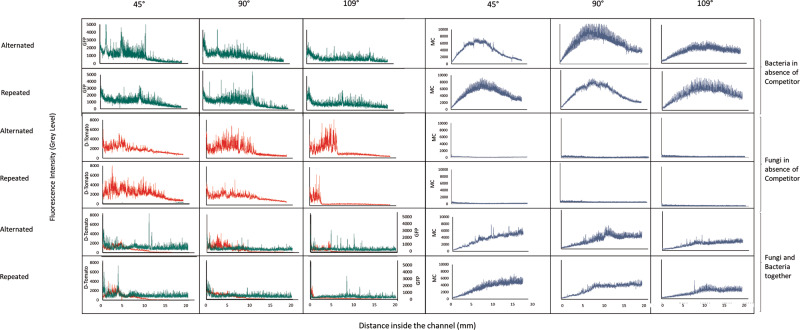
Examples of fluorescence profiles of *P. putida* expressing GFP constitutively (green), *C. cinerea* expressing d-Tomato constitutively (red), and 4-methylcoumarin (blue) inside the microfluidic channels. Profiles were obtained from the time point of highest fluorescence, day 2 for *P. putida* and day 6 for *C. cinerea* and 4-methylcoumarin, after inoculation inside the microfluidic channels. The fluorescence profiles show the changes in fluorescence along the length of every type of channel studied under the two competitor conditions. The beginning of the channel is located on the left side of each plot. The data from which these profiles were obtained is included in Supplementary Data 2.

Substrate degradation was distributed differently along the channels depending on the treatment conditions (Fig. 4), being highest in the middle of the channel and decreasing towards the ends for bacteria only. This effect was less strong in the sharper angled channels and changed to a gradual increase with increasing channel depth when fungi were present. Substrate degradation was negatively correlated with channel tortuosity only when bacteria and fungi grew together (Supplementary Fig. 4c, Supplementary Table 12).

## Discussion

Channel shapes similarly affected *C. cinerea* and *P. putida* when grown alone. When grown together, the effect of shapes on bacteria increased, whereas it remained similar on fungi. The pore space habitat inside the chip was dynamic and evolved over time as both strains grew. When both strains were together, fungi changed the physical environment so much that it resulted in a reduction in bacterial growth. Bacterial habitat modification, on the other hand, did not affect fungal foraging, which was only affected by the (solid) chip obstacles. The effect of the habitat modification was also reflected on the substrate consumption, which was affected by structures only when bacteria and fungi grew together.

Bacterial biomass was reduced as the turning angle became sharper. The effect of the angles was enhanced when their turn order was repeated. This interaction occurred, however, only at sharp angles, 90° and 109°. This result might be explained by the fact that a 45° turning angle is still inside the range of angles that *P. putida* tumble (randomly change direction) in a free-swimming environment^36^. Therefore, bacteria can swim through these channels without being significantly affected by the corners and the way they are arranged. On the other hand, in 90° and 109° angle channels, repeated turn order reduced the motility of bacteria more strongly. This suggests that bacteria not only keep a tumble frequency memory^37^ but also a tumble direction memory, which seems to be affected by the repeated turn order angles, leading to an increased time to cover a distance^38^. Bacterial biomass at 45°, however, was similar for both angle arrangements, suggesting that before the critical point (90°), turning directions do not affect the dispersal and growth of bacteria. Bacterial accumulation has been modeled to occur in 90° corners^39^ and similar accumulations seem to be occurring in the present experiment at 90° and 109°, which can block additional parts of the void pore space and by this impede further bacterial dispersal. It has been shown that the mean distance bacteria run before tumbling for bacterial strains such as *E. coli* is approximately 19 µm^40^ which is lower than the distance between channel turns in our experiment (50 µm). Having a shorter distance between the channel turns could, thus, produce a higher cell accumulation and thus a stronger effect on bacterial colonization of the channels.

The way structures affected bacterial biomass was stronger when fungi were present. In general, there was less bacterial biomass when cultivated together with the fungus. Since antibiotic production by *C. cinerea* has been reported to affect exclusively gram-positive bacteria^41^, we believe that the main reason for this must be competition for nutrients and space^42^.

Interestingly, when fungi were present, hyphae increased the effect that structures had on bacterial biomass, where sharper angles and repeated turn order reduced bacterial biomass more than when they were not present. Such findings, together with high magnification pictures (Fig. 1b), suggest that the habitat modification caused by fungi—by blocking the pore space with their hyphae—increased the complexity of the habitat where bacteria grew. Such blockages were not observed to be surpassed by bacteria during the duration of the experiment. In nature, fungi might act not only as bridges or networks^42,43^ and “highways” for bacterial dispersal^44,45^ but also as barriers that prevent bacteria from advancing further inside the soil pores. The interaction between the effect of structures and the presence of competitor in bacterial biomass indicates that competition does not only occur for nutrients but also for space.

Turns in the channels reduced fungal biomass as angles became sharper, the effect increased when turn order of the angles was repeated. Thus, the effect of an angle in a pore space depends on how it is positioned, similar to what has been suggested by models based on tomography images^35^. When fungal hyphae hit a wall, their Spitzenkörper shifts towards the wall and stays near the wall as hyphae grow^46^. Spitzenkörper seems to be responsible for the reorientation of hyphae after hitting an obstacle because it remains in the part of the hyphae that is close to the wall, in a phenomenon known as thigmotropism^47^ as a way to maintain its original directionality after the obstacle is circumvented. In alternated turns channels, hyphae seem to maintain the original directionality regardless of the contact angle reducing the effect of the angles on fungal biomass. The explanation can be as follows: once a hypha encounters an obstacle, the Spitzenkörper shifts towards the wall and stays near the wall as hyphae grow^46^ where it remains close to the wall, in line with the direction of its growth. When hitting the next wall in a channel with alternating turns, both directions would help hyphae to find the right path. This contrasts with channels with repeated turn order, where during every second turn, one of the two directions does not point towards the right path and instead hit a wall. Such findings can be corroborated by high-magnification analysis of single hyphal tip growth, showing that frequent hyphal branching occurred only at angles of 90° and 109° (Fig. 3, Supplementary Movies 1–6), and that hyphae suffered loss of direction in corners of repeated turn order (with 90° and 109°).

The fluorescence profiles (Fig. 4) show that the fungal biomass distribution along the channels was indeed affected by the angle sharpness. Hyphae covered longer distances in the 45° channels, but in the channels with angles of 90° and 109°, the fluorescence profile locally reached higher levels of fluorescence, specifically in the first parts of the channels. This could be explained by the fact that when hyphae hit a wall at an angle of 90° and 109°, the Spitzenkörper shrinks^46^ and leads to a branching event that initiates growth towards both sides. Eventually, one of the sides would reach the continuation of the path, whereas the other grows towards its origin or gets trapped between two corners. Such branching leads eventually to a localized accumulation of biomass in the beginning of the 90° and 109°-angled channels, even though their total biomass is lower than the 45°-angled channels (Figs. 3 and 4 and Supplementary Video 1–6). Branching events can be observed after hyphae encounter walls, but this does not generally stop the hyphal advancement in alternated turn orders as it was found for repeated turn order. The importance of branching for a successful fungal colonization in microstructures has been explored previously^48^; the present study suggests that the higher hyphal branching rates produced by encountering 90° and 109° corners lead to a different fungal biomass distribution along the different microstructures.

When growing with *P. putida*, the effect of structures on fungal growth did not change (Supplementary Fig. 5a, b). This suggests that there was minimal physical effect, such as clogging, caused by bacterial biomass, that could block the advancement of fungal hyphae. Moreover, high magnification movies (Supplementary Movies 1–6) showed that a bacterial blockage of a channel similar to the one produced by hyphae was not impeding fungal advancement in any of the channels.

Applied to a situation in real soil, this would mean that pore space passages forcing fungi to bend at a certain angle could be prone to cause a local accumulation of fungal biomass, where joint forces of several hyphal tips may support its effort to penetrate the pore wall. Models performed on soil derived from micro-computed tomography images consider that pore volume, pore connectivity, and presence of water bubbles affect fungal growth inside the pore space^35,49^. Although it remains to investigate exactly how much of a soil pore space can be protruded by fungal hyphae, the current study adds passage turning angle and the way they are arranged (turn order) to the characteristics that should be considered when evaluating the effect of soil architecture on fungal growth.

The effect that the studied angles had on fungi did not depend on the presence of bacteria but occurred similarly when the fungus grew alone. The same is observed in the linear regression of fungal biomass vs channel tortuosity in the absence and in the presence of bacteria (Supplementary Fig. 3, Supplementary Table 8). Therefore, the only effect of bacterial presence on fungal biomass seems to be an overall reduction of hyphae across all the angle profiles. This reduction can be mainly attributed to competition for nutrients. This seems to suggest that bacteria, as opposed to fungi, do not alter the spatial habitat in a way that would interfere with fungal foraging. Even though bacteria grow faster^50^ bacterial biomass did not change the effect structures had on fungal growth, as hyphae can easily push through bacterial colonies using protrusive forces^51,52^. Besides nutrient competition, other explanations of importance when studying polymicrobial interactions, such as quorum sensing signaling, which could change bacterial or fungal metabolisms^42^ might also play a role in the reduction of fungal biomass.

Our study confirms the reducing effect of acute angles on fungal space exploration found in ref. ^33,34^. Here, we increased the number of tested angles to include channels of acute, right, and obtuse angles and different directions, so that these principle spatial conditions can be further understood. Additionally, we also tested the effect of the turning order of the three angles as well as the effect of these structures on the presence of a bacterial strain. Being able to focus on fungal biomass via fluorescent markers instead of solely hyphal front progression allows us to evaluate fungal investment more carefully into different microstructures, and allows us to take into account the frequent biomass accumulations in corners of sharp turning angles.

Substrate degradation did not differ across angles and angle arrangements when bacteria and fungi grew by themselves. The two structure parameters only affected substrate degradation when bacteria and fungi grew together.

*P. putida* alone degraded the AMC through the different channels even though mineral nitrogen was provided. Regardless of the different biomass levels encountered in the channels, the substrate cleavage of the cells did not differ between channel type, which means that cells had a higher enzyme activity rate (Supplementary Table 13) in channels with sharper turning angle and repeated turn order. Fungi did not degrade the peptide when grown alone, which gives us the advantage that we could measure bacterial substrate degradation exclusively, even in the presence of the fungus. Since fungi have generally a higher nitrogen use efficiency than bacteria^53–55^, the mineral nitrogen provided in the medium could have sufficed for a much longer growth period than for bacteria. Nonetheless, *C. cinerea* did not show enzymatic activity that cleaves AMC in trials with an order of magnitude lower nitrogen levels.

When *C. cinerea* and *P. putida* were incubated together, the substrate degradation was different across angle and turn order types, following a similar pattern as bacterial and fungal biomass in those chips. While in co-cultured chips, bacterial and fungal biomasses were lower than those when they grew alone, indicating competition for nutrients, substrate degradation was higher in both channels with 45° angles. The increase of substrate consumption in low-angled channels may be caused by the onset of fungal peptide degradation under competition, an over-proportional increase in bacterial degradation under competition, or both. However, substrate degradation was lower in those with 109° angles compared to substrate degradation in chips with only one of the organisms (Fig. 3, Supplementary Fig 4c). The decreasing levels of substrate consumption in sharper-angled channels may partly be explained by the lowering biomass of both bacteria and fungi, but the same decrease in biomass did not affect substrate consumption in bacteria-only and fungi-only systems. Thus, the lowered substrate consumption in the 90° and 109°-angled channels is presumably due to the habitat modification produced by hyphae, as they block the channels and restrict passage for bacteria, and substrate and fluorophore exchange via diffusion between the channels and the pillar system. Finally, *P. putida* and *C. cinerea* seem unlikely to have been affected by additional biotic or abiotic stress since they were growing in a nutrient liquid medium, at room temperature, and a pH that is within the pH range for optimal growth of both strains. The two factors that are affecting both strains growth is either the physical constriction due to the chip structures, and the nutrient depletion that occurs over time. However, other factors that could produce stress, such as accumulation of metabolites or reduction of oxygen levels in the channels, were not measured but could potentially be parameters for future studies.

Our results show that the geometry of the pore space significantly affected how much bacteria and fungi grew. They also show that bacteria are affected by the habitat modification by fungal hyphae, whereas fungi were not affected by the habitat modification by bacteria. It has been suggested that spatial heterogeneity in substrate distribution can reduce respiration from soils^56^. In the present study, we do not see that structure itself could reduce substrate consumption of single cultures of bacteria, but the interaction of fungal biomass with structure allowed such a reduction to occur. This would mean, in a natural soil context, that a soil with a more intricate pore space structure including sharper angles would be less successfully explored than in a pore space dominated by less sharp turns, since it would force microbial communities to move or grow backwards to their original direction when exploring the soil pore space. Our results indicate that this is mainly due to the amount of energy for detours that needs to be invested in exploration, which is found to be higher in sharper angles. A reduction in the colonization of a pore space by microbial communities due to sharper angles would thus also lead to a reduction in organic matter turnover in these types of soils. Our experiment mainly revealed the effect of bacterial substrate consumption; hence, further experiments could address the factors influencing fungal degradation activity or focus on tracking how much of a common substrate is accessed by bacteria and fungi, and how much of it is assimilated into their biomass and consecutively into their necromass. To extrapolate our findings to the fate of organic matter in soil pore spaces there is the need to continue work with more diverse and more complex substrates that require subsequent attack by a wide variety of enzymes or radicals. However, our experiments address general effects of microstructures on fungal and bacterial growth, as well as bacterial enzymatic activity. Our findings highlight the fact that functions of different microbial groups can neither be assessed accurately when studied isolated without interactions, nor without considering the habitat where interactions occur, as brought forward in ref. ^57^. In the present study we can witness that microbial functions and competition can differ across space at micrometer scale, and can be influenced by habitat modification of the involved organisms.

Although real soil pore space physical parameters differ in more parameters than turning angle, this study succeeded in isolating this factor and showing how it influences the biomass distribution and substrate consumption of soil microbes. Further soil pore geometrical properties could be identified using the information provided from micro-computed tomography to include these parameters into microfluidic devices. In the present setup we used a saturated system where a defined liquid medium was filling the whole of the microfluidic device pore system, where both bacteria and substrates could freely move within the limits of the pore walls. In nature, under non-saturated conditions, water films alternate with air bubbles in soil pore spaces, hampering diffusion of substrates and creating barriers for swimming organisms^58–60^. Thus, air barriers and gas mobility should also be considered since they will likely influence outcomes of substrate availability. Also, the nutrients that microbes encounter in soil are commonly more patchily distributed, have a wider range of chemical complexity and fluctuate more over time than in our experiments where we used a defined medium. This variation in nutrients could interact with structures to enhance or inhibit microbial growth in the pore space. Therefore, experiments testing variation in nutrient proportions that mimic a variety of soil nutrient conditions could focus on studying interaction between nutrient conditions and structures on microbial growth. It would also be relevant to have a constant flow of nutrient medium inside the system, and thus avoid microbial starvation. It has been shown that when starvation initiates, the strength of pore clogging due to bacterial accumulation diminishes, increasing the permeability of the porous system^61^. Also, adding flows to the experimental system would elucidate how these structures affect the hydraulic properties of the bacterial colonization and possible biofilm formation. Flows and the location of the pores with respect to the flow have been shown to be crucial when predicting bacterial accumulation in porous systems^62,63^.

The habitat where microbes grow and interact is not stable over time, but it changes as a result of the microbial processes inside it. The need to better understand how microenvironments co-evolve with microorganisms has been pointed out previously^11,57^. In this study, we have only analyzed the interaction of two parameters of structure on the degradation of a specific substrate by two lab strains. We found that sharper angles and repeated turns reduced bacterial and fungal growth, as well as bacterial enzymatic activity. In nature, soils contain many more parameters of structure^64^, countless substrate types^65^, and countless microbial species interactions^66,67^. Despite the necessary simplifications, this study suffices to demonstrate the importance of microhabitats and to initiate a closer way of studying the complex role of physical structure on soil microbial ecology and on soil nutrient occlusion, leading ultimately to a better understanding of the laws of soil carbon storage. A deeper understanding of how microbes behave in microhabitats will give us the possibility to comprehend their role across all the environments in which they are present, from soil microbiology to gut microbiome and biofilm-forming pathogens.

## Materials and methods

### Chip design

**Fig. 5:**
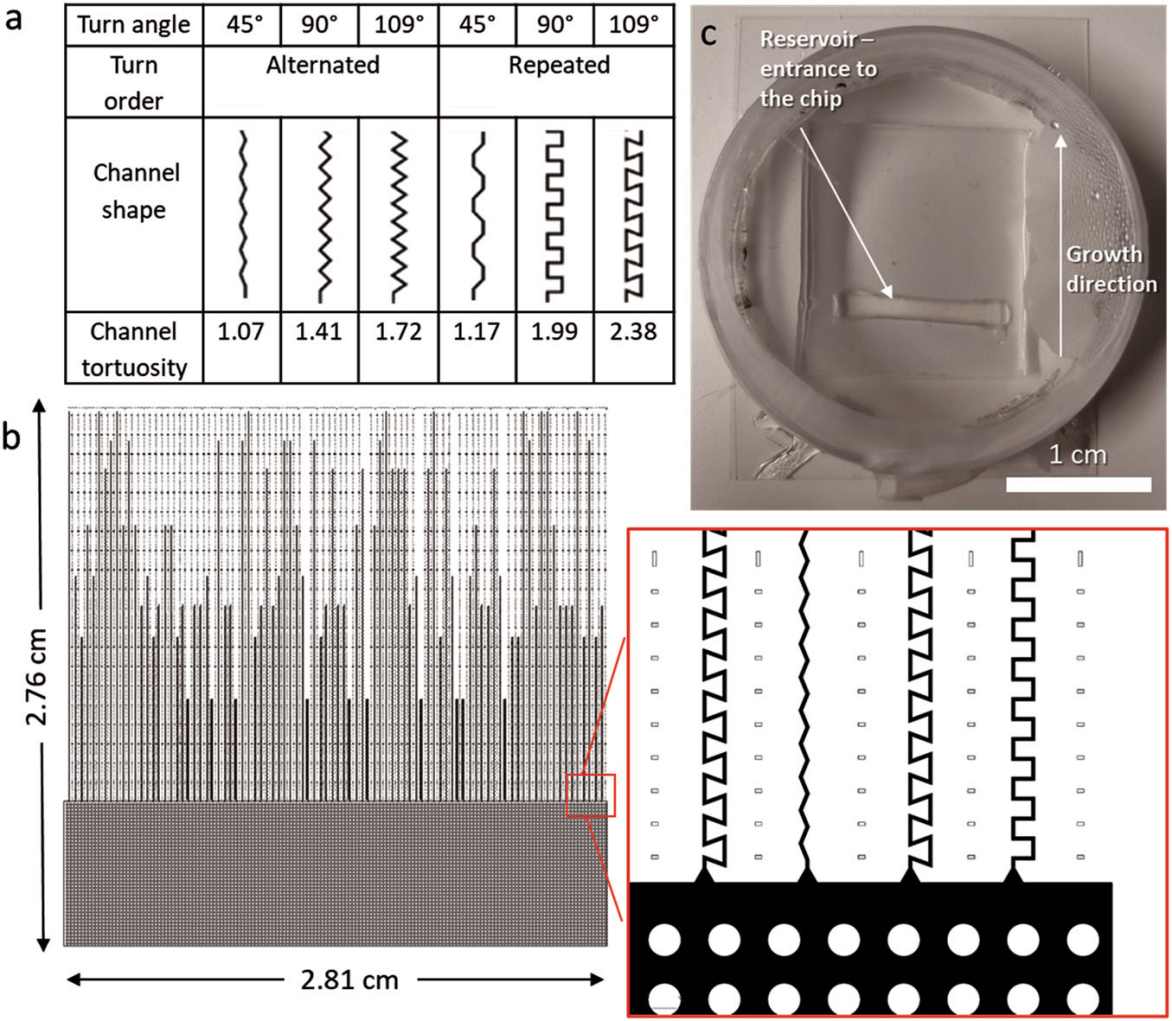
Chip design and arrangement of channels. **a** Channel geometries used in the Channel chip. Each channel is characterized by one type of bending angle (45°, 90°, and 109°) and one turn order (alternated or repeated). The channel tortuosity is shown in the last row, which is here quantified as the ratio between the distance inside the channel between two points and the distance between the same points in a straight line. **b** Chip design, consisting of a pillar system serving as the entry area to the channels, and the array of channels in six variations, randomly distributed along the chip. Structures in black correspond to the open areas of the microfluidic chip pore space in the channels and the pillar system. The chip design dimensions were: 281 mm × 276 mm and the volume of each channel was 2.42 nl. **c** The PDMS chip bonded to a custom-made glass bottom Petri dish, containing the growth medium and microbial cultures introduced via the rectangular reservoir opening.

The chip design was drawn in AutoCad 2018 (Autodesk) and consists of six kinds of treatment channels and a pillar system that served as an entrance to the channels (Fig. 5a, b). The pillar system is formed by pillars of 100 micrometers in diameter, separated by 100 micrometers, and allows bacteria and fungi to penetrate the full width of the chip before entering the treatment channels. The treatments consist of dead-end channels of six different geometries (*n* = 10, per chip) with the same internal volume. The channels are randomly distributed in parallel orientation along the chip.

The soil pore space in nature has a wide variety of physical parameters, such as the length of its pores, their diameter, and connectivity. In the present study we chose the approach of using abstract geometrical forms for formulation of general predictions of microbial responses to spatial structures, that in principle can be found within the broad spectrum of natural spatial pore space structures. The parameters assigned in this study were the “Angle” and “Turn order” of elongated pore channels. The angles used were 45˚, 90˚, 109˚, measured as the deviation from a continued straight line and thus the turning angle an organism in this channel needs to perform (Fig. 5a). In this way, we cover the three types of angles microorganisms might encounter in the soil pore space channels: obtuse, right, and acute. Channels of each angle had two types of arrangements, one with an alternated turn order, and one with a repeated turn order. Channel types with an alternated turn order followed a pattern of alternating right and left bends, while channels with repeated turn order followed a pattern of two right turns followed by two left turns. The channel lengths were adjusted so that every type of channel would contain the same volume (2.42 nl) with a width of 10 µm and a height of 12 µm. Each channel segment was 50 µm long before the next turn.

### Chip fabrication

The chip was molded in polydimethylsiloxane (PDMS) silicone rubber and bonded onto a glass bottom Petri dish^24^. The master that served as mold for the PDMS was in turn fabricated through photolithography using a photomask.

The photomask was made of soda lime glass with a thin layer of chromium (Nanofilm, CA, USA). The shapes were patterned with a dwl66 + mask writer (Heidelberg Instruments, Germany). A NdYag laser, 532 nm, was used to draw patterns on a photoresist, AZ1500. The pattern was subsequently developed in AZ 351B positive developer and the chromium etched in TechniEtchCr01 (Microchemicals GmbH, Ulm Germany). For master fabrication, SU-8 2015 (MicroChem, Newton, MA, USA) was dispensed onto a heat-dried (90 degrees 30 min) 3-inch silicon wafer (Siegert Wafer, Aachen, Germany) and spun at 4000 rpm to achieve a 12 µm thick layer. The SU-8 was exposed with UV-light in a contact mask aligner (Karl Suss MJB4 soft UV, Munich, Germany). After UV exposure, the non-crosslinked photoresist was developed (MrDev600) and rinsed with isopropanol. To prevent PDMS from sticking to the mold, the wafer was activated in oxygen plasma for 60 s (ZEPTO, Diener Plasma-Surface Technology, Germany) and exposed overnight to a vapor of trichloro (1H,1H,2H,2H-perfluorooctyl) silane (PFOTS, Sigma Aldrich, Saint Louis, MO, USA) at 180 degrees during which a monolayer is formed. SYLGARD^TM^ 184 PDMS (Dow Chemicals Company, Midland, Michigan) was made by mixing the elastomer with the curing agent in a mass proportion of 10:1, poured on top of the master, degassed at −15 kPa for one hour and polymerized in an oven at 60 °C for two hours.

The PDMS labyrinths were removed from the master and a rectangular portion of 2.5 cm × 0.5 cm was cut out in the middle of the pillar system, approximately 0.5 cm away from the entrance of every channel. This cut was made to create the reservoir that served as entrance to the labyrinth (Fig. 5c). The PDMS labyrinths and a glass bottom Petri dish were activated using a Zepto Plasma System (Diener Plasma Surface Technology, Germany; negative polarity; 1 min for cover slips and 10 s for PDMS labyrinths). Directly after activation, the surfaces were put together, forming a tight, irreversible bond^68^ and 150 μl of the M9 medium with the dissolved fluorogenic reporter was introduced through the reservoir. The liquid medium was pipetted into the chip entrance and dragged inside the chip structures by capillary forces. For the remainder of the experiment, no flow was induced and the liquid medium inside the chip was left to remain static, molecular movement is therefore expected to be dominated by diffusion.

### Bacterial strains and growth conditions

The fungal and the bacterial strain for this experiment were selected based on several criteria, namely the fact that they are naturally soil microorganisms, have both been widely studied, and have thus become model organisms for soil microbes. The bacterial strain used in the experiment was the gram-negative soil bacterium *Pseudomonas putida* mt-2 carrying plasmid-borne msfGFP-reporter constructs. The reason why this strain was chosen is because its natural habitat is soils, where it is found in high concentrations compared to other strains^69^. Also, its metabolism has been widely studied for bioremediation purposes^70–72^. Bacteria were pre-cultured overnight in M9 minimal medium (12.8 g/L NaHPO_4_.7H_2_O, 3 g/L KH_2_PO_4_, 0.5 g/L NaCl, 100 mg/L NH_4_Cl, 0.12 g/L MgSO_4_, 4 g/L d-Glucose, 11.66 mg/L CaCl_2_, 13.5 mg/L FeCl_2_, 125 mg/L MgCl_2_.6H_2_O, 1 mg/L MnCl_2_.4H_2_O, 1.7 mg of ZnCl_2_, 0.43 mg CuCl_2_.2H_2_O, 0.6 mg CoCl_2_.6H_2_O, 0.6 mg Na_2_MoO_4_.2H_2_O, pH 6.5)^73^ with pH 6.5 at 28 °C and agitated at 150 rpm.

The experiments with *P. putida* mt-2 were conducted as follows: 2 ml of overnight cultures were pelleted by centrifugation (5000 *g* for 10 min at 21 °C), and cells were resuspended in 0.5 ml of fresh M9 medium additionally containing L-Alanine 7-amido-4-methylcoumarin (AMC, 160 mg/L) to determine substrate consumption inside the chips. AMC is a fluorogenic substrate that becomes fluorescent when it is enzymatically hydrolyzed by aminopeptidase enzymes^74^. The fluorescence from the AMC shows the extent of enzymatic activity that the bacteria have, which can be quantified within each structure. 1.5 µl of the bacterial suspension was added to the entrance of the chip, previously filled with M9 + AMC medium, to obtain a final optical density at 600 nm of 0.2 OD_600_^75,76^.

### Fungal strains and growth conditions

The fungal strain used was *Coprinopsis cinerea* AmutBmut PMA412, expressing constitutively the cytoplasmic fluorescent dTomato protein^77^. *C. cinerea* is a naturally occurring soil litter decomposer which has been widely studied due to its facility to grow in define medium and be manipulated at any stage of its growth^78^. Also, it has been shown to produce promising results inside microfluidic devices. Pre-incubation was done in 1.5% agar plates containing Yeast Malt Glucose medium^32^. A rectangular plug of the mycelium sized 1 mm × 25 mm was placed upside down in the reservoir inside the chip. Care was taken to separate the fungal mycelium from the top of the agar plug so that no extra nutrients would be added to the medium. The inner part of the labyrinth was filled beforehand with the sterile M9 medium containing 160 mg AMC/L (pH 6.5) by capillary forces directly after bonding. After 48 h, once the hyphae had arrived at the entrance of the channels, the medium from the reservoir was extracted and replaced with sterile fresh medium. In the fungal-bacteria treatment, an inoculum of *P. putida* was introduced in the reservoir after the medium replacement to a final concentration of OD_600_ 0.2. This time point was marked as the start of the experiments containing fungi. Sterile wet tissues were placed inside the Petri dishes to preserve humidity. The plates were sealed with Parafilm to prevent water from evaporating and kept in the dark at room temperature (Fig. 5c). All the chips and the glass-bottomed Petri dishes for experiments with the bacterial and the fungal strain were previously sterilized under UV light in a sterile flow cabinet for 30 min. Every step that involved fungal or bacterial inoculation as well as the filling of the chips was done in a sterile flow cabinet. The nutrient medium inside the chips was not replaced after the start of the experiments.

In total, 15 chips were used for the experiment, 5 containing *Pseudomonas putida and* 5 with *Coprinopsis cinerea* (absence of competitor), and 5 containing both (presence of competitor).

### Microscopy

Epifluorescence microscopy was used for visualization of *P. putida, C. Cinerea*, and AMC using a fully motorized Nikon Ti2-E inverted microscope with PFS4 hardware autofocus, full 25 mm field-of-view, CoolLED pE300-White MB illumination connected via a 3 mm liquid light guide (LLG), and a Nikon Qi2 camera with 1x F-mount adapter. The filters used were LED-DAPI-A-2360A Semrock Filter Cube (Ex: 380–405 nm, Em: 413–480 nm), GFP-4050B Semrock Filter Cube (Ex: 444–488 nm, Em: 498–553 nm), mCherry-C Semrock Filter Cube (Ex: 520–585 nm, Em: 600–680 nm). The entire chip images for overall fluorescence quantification were captured using a (MRH00041) CFI Plan Fluor 4X, N.A. 0.13, W.D. 17.1 mm objective, with an exposure time of 20 ms for GFP, 100 ms for DAPI, and 100 ms for mCherry. For high magnification pictures a (MRD31905) CFI Plan Apochromat DM Lambda 100X Oil N.A. 1.45, W.D. 0.13 mm and a (MRD30405) CFI Plan Apochromat DM Lambda 40X, N.A. 0.95, W.D. 0.21 mm objective were used. NIS-Elements software was used for coordination of the multipoint imaging. Pictures were taken for every chip for 14 days. The days selected for analysis were the ones of maximum biomass, namely day 2 for *Pseudomonas putida* biomass and its AMC consumption, and day 6 for *Coprinopsis cinerea* biomass, its AMC consumption, and the AMC consumption of the fungal+bacterial conditions.

### Image analysis

The fluorescence intensity was quantified using ImageJ 1.52n^79^. Background was subtracted using the ImageJ rolling ball algorithm^80^ using 7 pixels as radius of rolling ball for images taken with 4X objective. The rolling ball radius was given based on the size of the biggest fluorescent object, which was the width of a channel. After the subtraction, the mean florescence intensity per pixel was quantified inside each channel using the ROI manager tool. The rectangular ROIs were of the same size and covered every individual channel of the experiment.

To attain a deeper understanding of the fluorescence distribution along the channels, fluorescent profiles were obtained for every type of channel. For this purpose, the segmented line tool and the measure tool were used to cover manually the entire length of the channels.

Besides the images obtained with the 4X objective, the 40X and the 100X objectives were used to obtain higher magnification images to facilitate overall result interpretation. The time-lapse movies of colonizing hyphae were obtained with a 40X objective every 5 min for a total period of 12 h.

### Statistics and reproducibility

For in-depth statistical analysis, the data from the day typically showing maximum biomass (indicated by maximum fluorescence, Supplementary Fig. 1) was chosen, namely day 2 for bacteria and day 6 for fungi. Day 6 was also selected for substrate consumption comparison.

The experiment had a full-factorial design with the factors angle (45°, 90°, 109°), turn order (alternated or repeated), and competition (presence or absence of the other organism). Each chip had 10 channels of each type (with all the angle-turn order combinations), and five chips of each inoculation type were analyzed: 5 with bacteria (bacteria in the absence of competitor), 5 with fungi (fungi in the absence of competitor), and 5 with bacteria and fungi (presence of competitor). Each channel was treated as a single replicate, meaning, 10 replicates of each type of channel in each chip. Multilevel model fitting correcting for random effects was used to test the influence of every factor on the variables. Additionally, a two-tailed three-way multivariate ANOVA correcting for random effects was conducted using R^81^ for testing significant differences in variances. Random effects were attributed to each microfluidic device as a physical replicate of the experiment. Angle, turn order, and competition were considered fixed factors for bacterial and fungal biomass, while angle, turn order, and organism (fungi, bacteria, fungi+bacteria) were the fixed factors for substrate consumption. Fluorescence data was log-transformed to obtain normality of the residuals and homogeneity of variances. The significance threshold used for all statistical tests was *p* < 0.05. When significant differences were found in the ANOVA, interactions were analyzed separately using Dunn’s method for multiple comparison of means^82^. Pairwise comparisons were performed with *t*-tests, using Holm corrected *p*-values^83^.

Also, linear regressions were done using R, with bacterial biomass, fungal biomass, and substrate consumption as dependent variables and tortuosity of the channels as independent variables.

### Reporting summary

Further information on research design is available in the Nature Research Reporting Summary linked to this article.

## Supplementary information

Transparent Peer Review File

Supplementary Information

Description of Additional Supplementary Files

Supplementary Videos 1-6

Supplementary Data 1

Supplementary Data 2

Reporting Summary

## Data Availability

The raw image data that support the findings of this study are openly available in Dryad at 10.5061/dryad.prr4xgxmw (part 1) and 10.5061/dryad.2fqz612pv (part 2)^84,85^.
