# Supplementary material for: Habitat geometry in artificial microstructure affects bacterial and fungal growth, interactions, and substrate degradation
Source: Commun Biol. 2021 Oct 26;4:1226. doi: 10.1038/s42003-021-02736-4 (PMC8548513; doi:10.1038/s42003-021-02736-4)
Supplement: Supplementary file 2 — Supplementary Information [file 42003_2021_2736_MOESM2_ESM.pdf]

SUPLEMENTARY MATERIAL

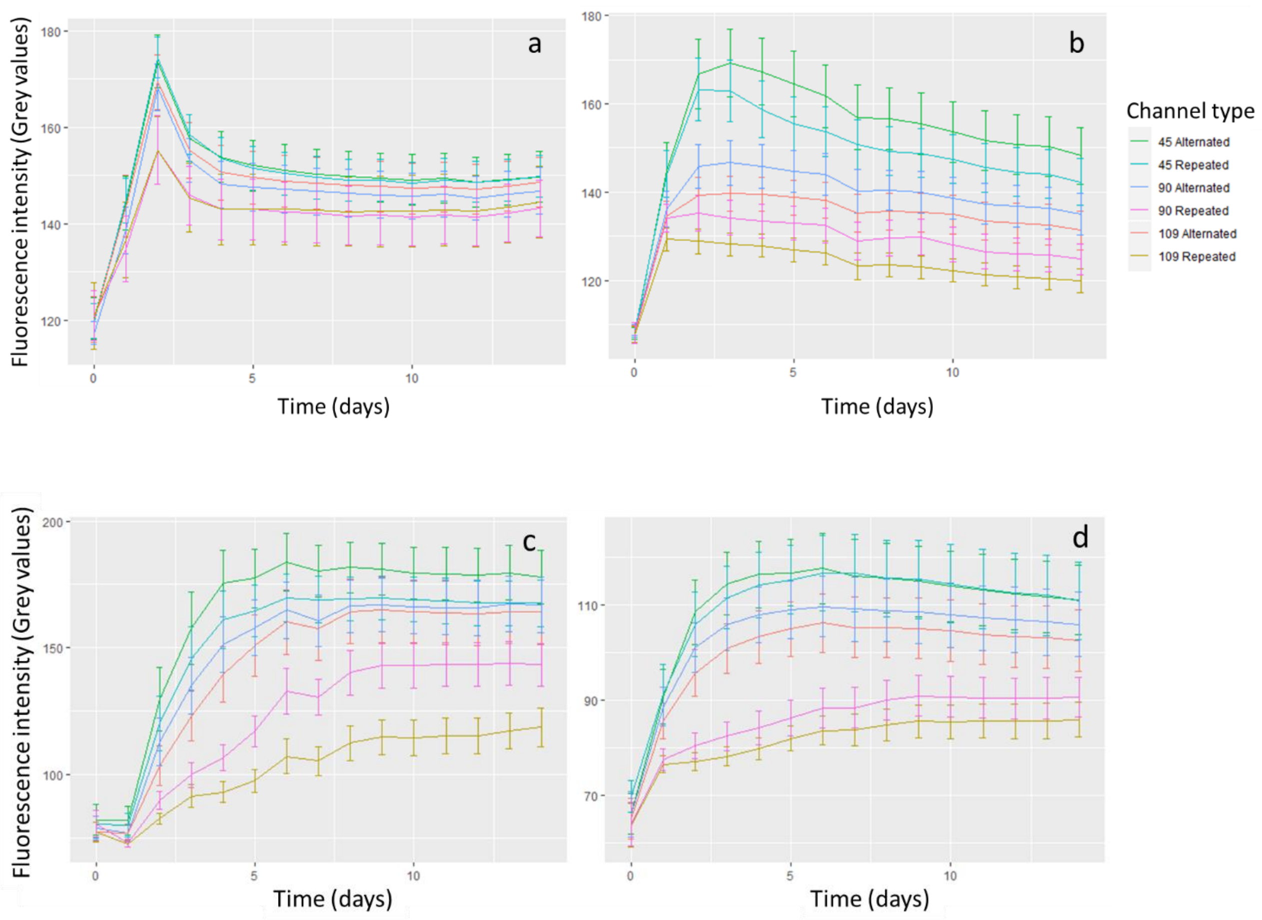

Supplementary Figure 1. *Bacterial biomass of* Pseudomonas putida *estimated via its constitutive expression of GFP inside the microfluidic channels, during the 14 days after inoculation, growing without (a), and with the competitor* Coprinopsis cinerea *(b). The Y-axis represents the fluorescence intensity, quantified as grey value, of GFP corresponding to each type of channel (n=50).* Fungal biomass of *Coprinopsis cinerea* expressing d-Tomato constitutively inside the microfluidic channels, during the 14 days after inoculation, growing without (c), and with the competitor *Pseudomonas putida* (d). The Y-axis represents the fluorescence intensity of d-Tomato corresponding to each type of channel and error bars indicater standard error (*n*=50).

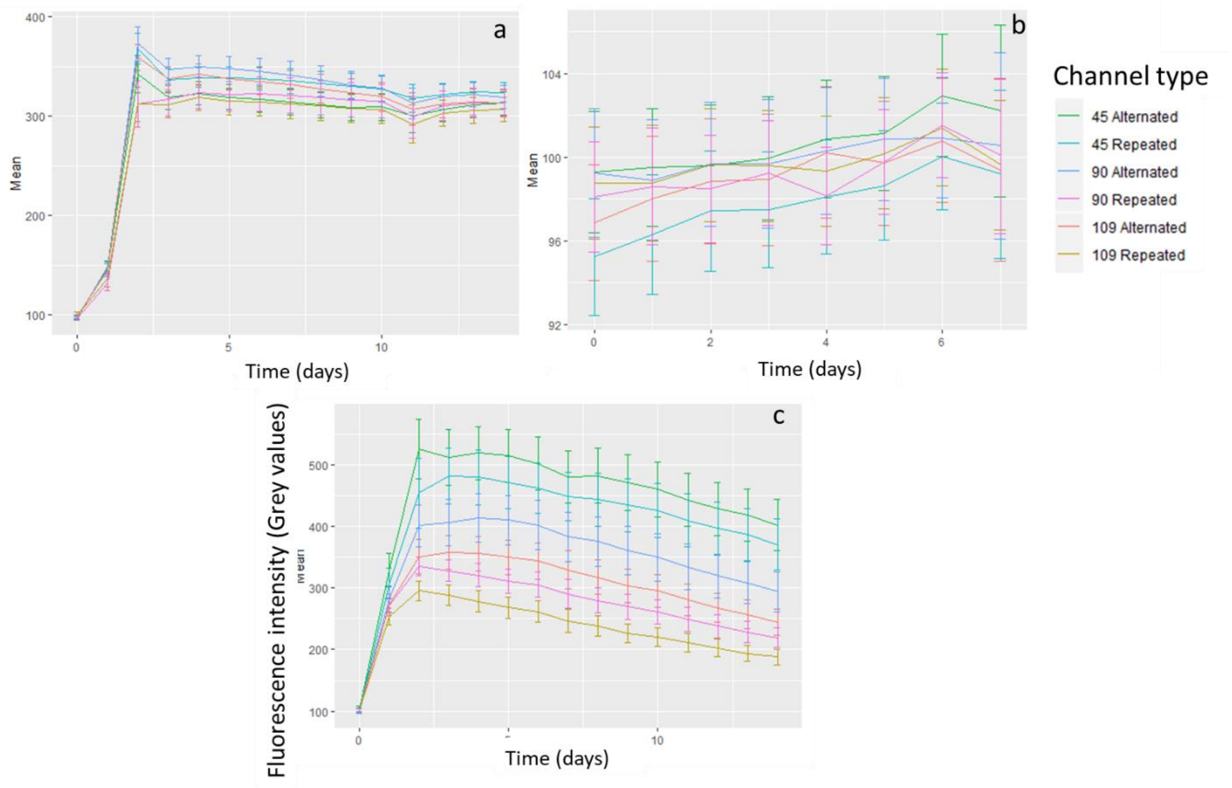

Supplementary Figure 2. Substrate consumption of L-Alanine 7-amido-4-methylcoumarin trifluoroacetate salt inside the microfluidic channels, during the 14 days after inoculation. The panels show bacterial experiment with *Pseudomonas putida* (a), *Coprinopsis cinerea* (b) and together both organisms together (c). The Y-axis represents the fluorescence intensity of 4-methylcoumarin corresponding to each type of channel and error bars indicater standard error (*n*=50).

	Sum of squares	Mean Square	Degrees of freedom	Density degrees of freedom	F value	P value	
Angle	1.797	0.899	2	580	98.737	<2.2e-16	***
Turn order	0.328	0.328	1	580	36.028	3.43e-09	***
Competition	0.104	0.104	1	8	11.409	0.01	**
Angle*Turn order	0.212	0.106	2	580	11.627	1.12e-05	***
Angle*Competition	0.279	0.14	2	580	15.332	3.25e-07	***
Competition*Turn order	0.002	0.002	1	580	0.249	0.618	
Angle*Competition*Turn order	0.0004	0.0002	2	580	0.024	0.977	
**Angle*Turn order**							
Angle @Alternated TO	0.388	0.194	2	288	2.066	0.09962	.
Angle @Repeated TO	1.620	0.810	2	288	8.618	1.1e-4	***
**Angle*Competition**							
Angle @Absence	0.343	0.172	2	293	1.829	0.1406	
Angle @Presence	1.732	0.866	2	293	9.226	7e-5	*

Pairwise comparison:

**Turn order = Repeated TO**	45	90
90	4.3e-11	-
109	4.2e-15	0.14

**Competition = Presence**	45	90
90	6.1e-14	-
109	<2e-16	0.012

Supplementary Table 1. Output of three-way ANOVA of the variable log-transformed bacterial biomass measured via their GFP fluorescence, with chip as random effect and Angle, Turn order, and Competition as fixed factors. Contrasts Angle*Turn order and Angle*Competition were analyzed separately using the Dunn’s method for multiple comparison of means. Pairwise comparisons were done with t-tests with p values adjusted using Holm corrections[58].

Model	*df*	*AIC*	*BIC*	*logLik*	*Test*	*L.Ratio*	*p-value*
1	3	-840.1047	-826.9139	423.0524			
Angle	5	-988.4581	-966.4735	499.2291	152.35341	1vs2	<.0001
Turn order	6	-1019.0571	-992.6755	515.5285	32.59893	2vs3	<.0001
Competition	7	-1025.9199	-995.1414	519.9599	8.86280	3vs4	0.0029
*Angle*Competition*	9	-1051.1584	-1011.5861	534.5792	29.23858	4vs5	<.0001
*Turn order*Competition*	10	-1049.4017	-1005.4324	534.7008	0.24321	5vs6	0.6219
*Angle*Turn order*	12	-1015.8292	-1068.5924	546.2962	23.19071	6vs7	<.0001
*Angle*Turn order*Competition*	14	-1064.6408	-1003.0837	546.3204	0.04839	7vs8	0.9761

Supplementary Table 2. Output of the multi-level model fitting for the variable log-transformed bacterial biomass measured via their GFP fluorescence, with chip as random effect and Angle, Turn order, and Competition as fixed factors. Each step performs an ANOVA and compares the model with the previous model.

	Value	*Std.Error*	*DF*	*t-value*	*p-value*
(Intercept)	5.072999	0.033199	582	152.8039	0
Ang 45 – Ang 90	0.105983	0.016518	582	6.41606	0
Ang 109 - Ang 90	-0.01035	0.016518	582	-0.62646	0.5313
Alternated TO – Repeated TO	0.070932	0.015574	582	4.5546	0
Presence – Absence	-0.17115	0.046301	8	-3.69643	0.0061
Ang 45 – 90 (Pres-Abs)	0.065965	0.019074	582	3.45839	0.0006
Ang 109 – 90 (Pres – Abs)	-0.03847	0.019074	582	-2.01675	0.0442
Alternated TO – Repeated TO (Pres – Abs)	-0.00777	0.015574	582	-0.49879	0.6181
Ang 45 – 90 (Alternated TO – Repeated TO)	-0.072955	0.019074	582	-3.8249	0.0001
Ang109 – 90 (Alternated TO – Repeated TO)	0.012044	0.019074	582	0.63144	0.528

Supplementary Table 3. Contrasts of the multi-level model with the variable log-transformed bacterial biomass measured via their GFP fluorescence, with chip as random effect and Angle, Turn order, and Competition as fixed factors.

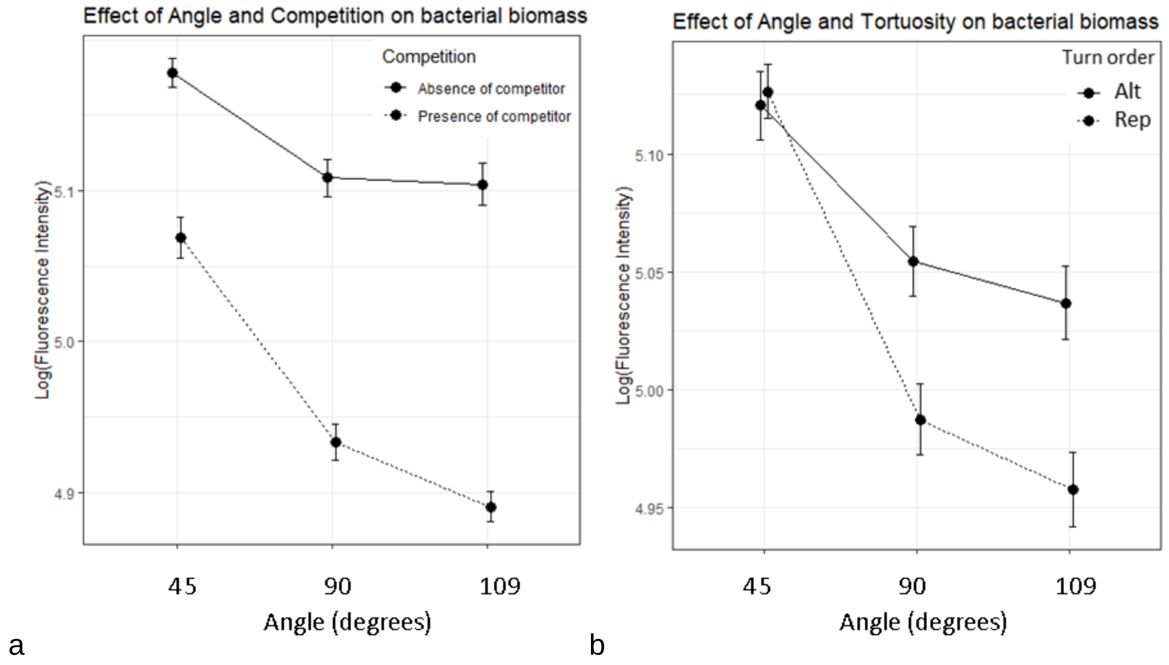

Supplementary Figure 3. Effect of interactions Angle-Competition (a) and Angle-Turn order (b) on the *Pseudomonas putida* biomass measured via their GFP fluorescent signal. The fluorescence data are log-transformed and presented for the alternated (continuous line) and for repeated turn order (doted line). The points represent the mean log-transformed fluorescence for each treatment and the error bars represent the ±standar error based on ANOVA for all the angles (*n*=50).

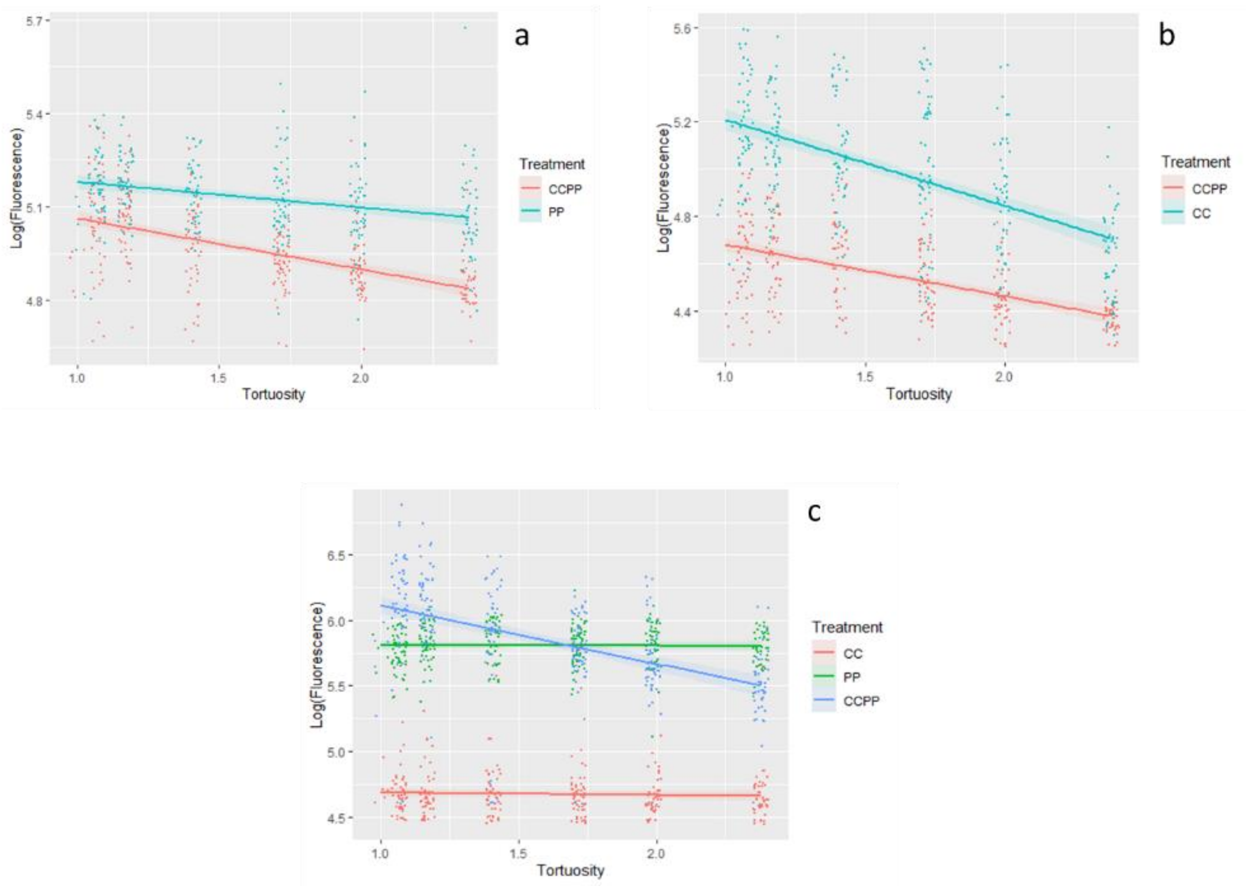

Supplementary Figure 4*.* The effect of tortuosity on bacterial biomass (a), fungal biomass (b) and substrate consumption (c), shown as linear correlations. The Y-axis shows the log transformed value of fluorescence intensity of GFP for bacteria, d-Tomato for fungi, and 4-methylcoumarin for the substrate. Different colours represent the experimental conditions: PP for bacteria only, CC for fungi only, and CCPP for bacteria and fungi growing together. The scattered points represent single measurements of fluorescence (*n*=50). Shaded areas represent confidence intervals.

	Value	Std.Error	DF	t-value	p-value
(Intercept)	5.227055	0.040156	588	130.1694	<1E-10
Tortuosity	-0.163643	0.012562	588	-13.02687	<1E-10
Presence-Absence	0.035249	0.056789	8	0.6207	0.5521
*Tor*Presence - Tor*Absence*	0.081356	0.017765	588	4.57949	<1E-10
Absence					
(Intercept)	5.262304	0.046053	294	114.26531	<1E-10
Tortuosity (slope)	-0.082287	0.010905	294	-7.54597	<1E-10
Presence					
(Intercept)	5.227055	0.033227	294	157.31152	<1E-10
Tortuosity (slope)	-0.163643	0.014025	294	-11.66821	<1E-10

Supplementary Table 4. Output of the linear model with bacterial biomass (GFP fluorescence signal) as dependent variable and channel tortuosity as independent variable. Second and third panel indicate linear regression for bacterial biomass data in absence and presence of competitor respectively.

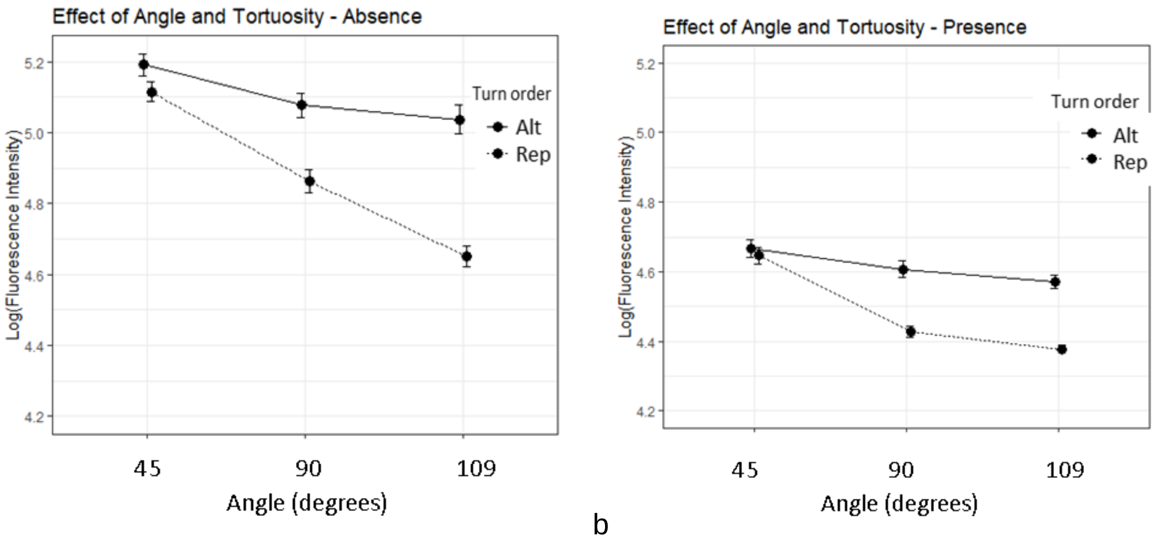

a b

Supplementary Figure 5. Effect of interactions Angle-Turn order in absence of competitor (a) and in presence of competitor (b) on the *Coprinopsis cinerea* biomass measured with d-Tomato fluorescent signal. The fluorescence data are log-transformed and presented for the alternated (continuous line) and for the repeated turn order (doted line). The points represent the mean log-trasnformed fluorescence for each treatment and the error bars represent the ±standard error based on ANOVA for all the angle types (*n*=50).

	Sum of squares	Mean Square	Degrees of freedom	Density degrees of freedom	F value	P value	
Angle	6.236	3.118	2	580	148.188	<2.2e-16	***
Turn order	4.762	4.762	1	580	226.344	<2.2e-16	***
Competition	0.497	0.497	1	8	23.603	0.001	**
Angle*Turn order	1.484	0.742	2	580	35.254	3.55e-15	***
Angle*Competition	0.418	0.209	2	580	9.928	5.76e-15	***
Competition* Turn order	0.332	0.332	1	580	15.766	8.07e-05	***
Angle*Competition* Turn order	0.183	0.091	2	580	4.345	0.013	*
**Competition**							
Angle*Turn order @Presence	0.464	0.232	2	290	1.627	0.18963	
Angle*Turn order @Absence	1.202	0.601	2	290	4.209	0.00613	**
**Competition = Absence**							
Angle @ Turn order = Repeated	5.398	2.699	2	143	18.9	<1e-6	***
Angle @ Turn order = Alternated	0.641	0.321	2	143	2.245	0.07670	.
**Competition = Presence**							
Angle @ Turn order = Repeated	2.048	1.024	2	143	7.17	0.00634	***
Angle @ Turn order = Alternated	0.233	0.116	1	143	0.817	0.6641	

Pairwise comparisons:

**Competition= Absence Turn order = Repeated TO**	45	90
90	3.8e-8	-
45	<2e-16	1.7e-06

**Competition = Presence**	45	90
90	7.2e-9	-
45	1.3e-13	0.065

Supplementary Table 5. Output of three-way ANOVA of the variable *Coprinopsis cinerea* log transformed biomass measured with d-Tomato fluorescence, with chip as random effect and Angle, Turn order, and Competition as fixed factors. Contrasts were analyzed separately using Dunn's method for multiple comparison of means. Pairwise comparisons were done with t-tests with p values adjusted using Holm corrections[58]. The significance of the three way interaction is likely to be produced because a similar interaction of angle and turn order in

presence of bacteria could not be similar to the same interaction in absence of bacteria since fungal biomass levels are already showing a minimum growth, close to background levels.

	Model	*df*	*AIC*	*BIC*	*logLik*	*Test*	*L.Ratio*	*p-value*
1	1	3	-128.47	-115.28	67.23513			
Angle	2	5	-289.05	-267.066	149.5251	1 vs 2	164.5798	<.0001
Turn order	3	6	-453.421	-427.04	232.7106	2 vs 3	166.3711	<.0001
Competition	4	7	-465.159	-434.381	239.5797	3 vs 4	13.73815	0.0002
*Angle*Competition*	5	9	-478.266	-438.694	248.1331	4 vs 5	17.10689	0.0002
*Turn order*Competition*	6	10	-490.211	-446.242	255.1056	5 vs 6	13.9449	0.0002
*Angle*Turn order*	7	12	-552.955	-500.192	288.4775	6 vs 7	66.7438	<.0001
*Angle*Turn order*Competition*	8	14	-557.729	-496.172	292.8645	7 vs 8	8.77419	0.0124

Supplementary Table 6. Output of the multi-level model fitting for the variable log-transformed fungal biomass measured with d-Tomato fluorescence, with chip as random effect and Angle, Turn order, and Competition as fixed factors. Each step performs an ANOVA and compares the model with the previous model.

	Value	*Std.Error*	*DF*	*t-value*	*p-value*
(Intercept)	5.077005	0.060732	580	83.5974	0
Ang 45 – Ang 90	0.113564	0.029056	580	3.90849	0.0001
Ang 109 - Ang 90	-0.04097	0.029056	580	-1.41019	0.159
Alternated TO – Repeated TO	-0.21461	0.029056	580	-7.38624	0
Presence – Absence	-0.47	0.085887	8	-5.47231	0.0006
Ang 45 – 90 (Pres-Abs)	-0.05389	0.041091	580	-1.31138	0.1902
Ang 109 – 90 (Pres – Abs)	0.005047	0.041091	580	0.12282	0.9023
Alternated TO – Repeated TO (Pres – Abs)	0.034485	0.041091	580	0.83924	0.4017
Ang 45 – 90 (Alternated TO – Repeated TO)	0.13887	0.041091	580	3.37959	0.0008
Ang109 – 90 (Alternated TO – Repeated TO)	-0.17066	0.041091	580	-4.15322	0
Ang 45 – 90 (Alternated TO – Repeated TO) (Pres – Abs)	0.021144	0.058111	580	0.36385	0.7161
Ang109 – 90 (Alternated TO – Repeated TO) (Pres – Abs)	0.157559	0.058111	580	2.71134	0.0069

Supplementary Table 7. Contrasts of the multi-level model with the variable log-transformed fungal biomass measured with d-Tomato fluorescence, with chip as random effect and Angle, Turn order, and Competition as fixed factors.

	Value	*Std.Error*	*DF*	*t-value*	*p-value*
(Intercept)	5.572636	0.07139	588	78.05873	<1E-10
Tortuosity	-0.363774	0.019788	588	-18.38381	<1E-10
Presence-Absence	-0.6736	0.100961	8	-6.67188	2.00E-04
*Tor*Presence - Tor*Absence*	0.145963	0.027984	588	5.21595	<1E-10
Absence					
(Intercept)	5.572636	0.088027	294	63.30601	<1E-10
Tortuosity (slope)	-0.363774	0.02319	294	-15.68649	<1E-10
Presence					
(Intercept)	4.899036	0.049441	294	99.08868	<1E-10
Tortuosity (slope)	-0.21781	0.015663	294	-13.90632	<1E-10

Supplementary Table 8. Output of the linear model with fungal biomass (d-Tomato fluorescence signal) as dependent variable and channel tortuosity as independent variable.

Second and third panel indicate linear regression for fungal biomass data in absence and presence of competitor respectively.

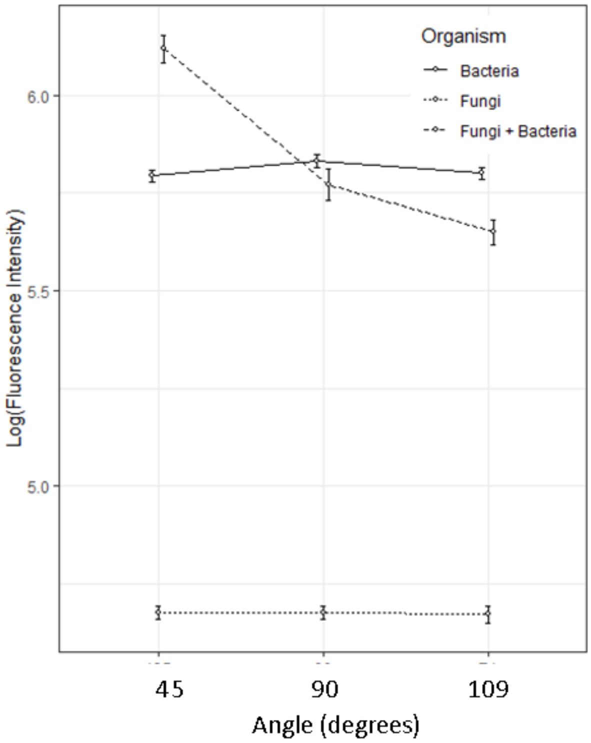

Supplementary Figure 6. Effect of the interaction between the factors Angle and Organism on the substrate consumption. The 4-methylcoumarin fluorescence data are presented for the bacterial *Pseudomonas putida* (continuous line), fungal *Coprinopsis cinerea* (doted line), and fungal-bacterial (dashed line) experiments. The points represent the mean fluorescence for each treatment and the error bars represent the ±standard error based on an ANOVA for all angle types (n=50).

	Sum of squares	Mean Square	Degrees of freedom	Density degrees of freedom	F value	P value	
Angle	3.773	1.886	2	870	43.934	<2.2e-16	***
Turn order	0.788	0.788	1	870	18.351	2.04e-05	***
Organism	8.794	4.397	2	12	102.403	2.88e-08	***
Angle*Turn order	0.700	0.350	2	870	8.157	3.09e-04	***
Angle*Organism	8.243	2.061	4	870	47.993	<2.2e-16	***
Organism*Turn order	0.983	0.491	2	870	11.442	1.25e-05	***
Angle*Organism*Turn order	0.303	0.076	4	870	1.765	0.134	
**Angle*Turn order**							
Angle @Alternated	0.642	0.321	2	433	2.441	>0.05	
Angle @Repeated	3.831	1.916	2	433	14.53	<0.01	*
**Angle*Organism**							
Angle @Bacteria	0.081	0.041	2	293	0.309	>0.05	
Angle @Fungi	0.001	6.7e-4	2	293	5.12e-3	>0.05	
Angle @Bacteria + Fungi	11.932	5.966	2	293	45.39	<0.01	*
**Turn order*Organism**							
Turn order @Bacteria	8.4e-3	8.4e-3	1	294	0.064	>0.05	
Turn order @Fungi	0.015	0.015	1	294	0.117	>0.05	
Turn order @Bacteria + Fungi	1.747	1.747	1	294	13.28	<0.01	*

Pairwise comparison:

**Turn order = Repeated**	45	90
90	0.039	-
45	3.3e-03	0.348

**Organism = Bacteria + fungi**	45	90
90	1e-10	-
45	<2e-16	0.018

Supplementary Table 9. Output of three-way ANOVA of the variable substrate consumption, measured with 4-Methylcoumarin fluorescence, with chip as random effect and Angle, Turn order, and Organisms as fixed factors. Contrasts were analyzed separately using the Dunn’s method for multiple comparison of means. Pairwise comparisons were done with t-tests with p values adjusted using Holm corrections.

	Model	df	AIC	BIC	logLik	Test	L.Ratio	p-value
1	1	3	98.14038	112.5476	-46.0702			
Angle	2	5	35.67437	59.68634	-12.8372	1 vs 2	66.46601	<.0001
Turn order	3	6	23.14013	51.9545	-5.57007	2 vs 3	14.53424	0.0001
Organism	4	8	-16.2714	22.14778	16.13569	3 vs 4	43.41151	<.0001
*Angle*Organism*	5	12	-176.617	-118.988	100.3084	4 vs 5	168.3455	<.0001
*Turn order*Organism*	6	14	-195	-127.767	111.5002	5 vs 6	22.38354	<.0001
*Angle*Turn order*	7	16	-207.31	-130.472	119.6551	6 vs 7	16.30971	0.0003
*Angle*Turn order*Organism*	8	20	-206.461	-110.413	123.2304	7 vs 8	7.15064	0.1281

Supplementary Table 10. Output of the multi-level model fitting for the variable log-transformed substrate consumption measured with 4-Methylcoumarin fluorescence, with chip as random effect and Angle, Turn order, and Organism as fixed factors. Each step performs an ANOVA and compares the model with the previous model.

	Value	Std.Error	DF	t-value	p-value
(Intercept)	5.851698	0.063653	874	91.93076	0
Ang 45 – Ang 90	-0.09126	0.03395	874	-2.68804	0.0073
Ang 109 – Ang 90	-0.02163	0.03395	874	-0.63701	0.5243
Repeated TO – Alternated TO	-0.03984	0.030992	874	-1.28549	0.199
Fungi – Bacteria	-1.15348	0.088946	12	-12.9683	0
Fungi+Bacteria – Bacteria	0.010437	0.088946	12	0.11735	0.9085
Ang 45 – Ang 90 (Fungi – Bacteria)	0.035737	0.04158	874	0.85948	0.3903
Ang 109 – Ang 90 (Fungi -Bacteria)	0.026321	0.04158	874	0.63301	0.5269
Ang 45 – Ang 90 (Fungi+Bacteria – Bacteria)	0.386306	0.04158	874	9.29061	0
Ang 109 – Ang 90 (Fungi+bacteria – Bacteria)	-0.0905	0.04158	874	-2.17648	0.0298
Repeated TO – Alternated TO (Fungi – Bacteria)	-0.00368	0.03395	874	-0.10841	0.9137
Repeated TO – Alternated TO (Fungi+Bacteria – Bacteria)	-0.14198	0.03395	874	-4.18213	0
Ang 45 – Ang 90 (Repeated TO – Alternated TO)	0.107305	0.03395	874	3.16066	0.0016
Ang 109 – Ang 90 (Repeated TO – Alternated TO)	-0.01965	0.03395	874	-0.57869	0.5629

Supplementary Table 11. Contrasts of the multi-level model with the variable log-transformed substrate consumption measured with 4-Methylcoumarin fluorescence, with chip as random effect and Angle, Turn order, and Organism as fixed factors.

	Value	*Std.Error*	*DF*	*t-value*	*p-value*
(Intercept)	4.705342	0.078689	882	59.79673	<1E-10
Bacteria – Fungi	1.11153	0.111283	12	9.98832	<1E-10
Bacteria + Fungi - Fungi	1.857722	0.111283	12	16.69367	<1E-10
Tortuosity	-0.019443	0.026835	882	-0.72454	0.4689
B:Tortuosity – F:Tortuosity	0.014389	0.03795	882	0.37917	0.7047
B+F:Tortuosity – F:Tortuosity	-0.426684	0.03795	882	-11.24339	<1E-10

Supplementary Table 12. Output of the linear model with substrate consumption (4-methylcoumarin fluorescence signal) as dependent variable and organism present and channel tortuosity as independent variables.

Turn Order	*Alternated*			*Repeated*		
Angle	45	90	109	45	90	109
Ratio Log (Substrate degradation fluorescence)/ Log (B GFP Fluorescence)	1.113	1.138	1.132	1.125	1.145	1.14

Supplementary Table 13. Output of the linear model with substrate consumption (4-methylcoumarin fluorescence signal) as dependent variable and organism present and channel tortuosity as independent variables.
