# Supplementary material for: Habitat geometry in artificial microstructure affects bacterial and fungal growth, interactions, and substrate degradation
Source: Commun Biol. 2021 Oct 26;4:1226. doi: 10.1038/s42003-021-02736-4 (PMC8548513; doi:10.1038/s42003-021-02736-4)
Supplement: Supplementary file 3 — Description of Additional Supplementary Files [file 42003_2021_2736_MOESM3_ESM.pdf]

Description of Additional Supplementary Files

File name: Supplementary Videos 1-6

Description:

Supplementary Video 1: Timelapses (every 5 min) of the hyphal growth of Coprinopsis cinerea inside the channel with 45° turning angle and alternated turn order.

Supplementary Video 2: Timelapses (every 5 min) of the hyphal growth of Coprinopsis cinerea inside the channel with 90° turning angle and alternated turn order.

Supplementary Video 3: Timelapses (every 5 min) of the hyphal growth of Coprinopsis cinerea inside the channel with 109° turning angle and alternated turn order.

Supplementary Video 4: Timelapses (every 5 min) of the hyphal growth of Coprinopsis cinerea inside the channel with 45° turning angle and repeated turn order.

Supplementary Video 5: Timelapses (every 5 min) of the hyphal growth of Coprinopsis cinerea inside the channel with 90° turning angle and repeated turn order.

Supplementary Video 6: Timelapses (every 5 min) of the hyphal growth of Coprinopsis cinerea inside the channel with 109° turning angle and repeated turn order.

File name: Supplementary Data 1

Description: Corresponds to the fluorescence data used to obtain Figure 2 and 3. These data was also used for all the statistical analysis.

File name: Supplementary Data 2

Description: Corresponds to the fluorescence data along the channels of the chip, used to plot Figure 5.
