# Supplementary material for: Habitat geometry in artificial microstructure affects bacterial and fungal growth, interactions, and substrate degradation
Source: Commun Biol. 2021 Oct 26;4:1226. doi: 10.1038/s42003-021-02736-4 (PMC8548513; doi:10.1038/s42003-021-02736-4)
Supplement: Supplementary file 7 — Reporting Summary [file 42003_2021_2736_MOESM7_ESM.pdf]

nature research

Corresponding author(s): Carlos Arellano-Caicedo

Last updated by author(s): 26/08/21

Reporting Summary

Nature Research wishes to improve the reproducibility of the work that we publish. This form provides structure for consistency and transparency in reporting. For further information on Nature Research policies, see our Editorial Policies and the Editorial Policy Checklist.

Statistics

For all statistical analyses, confirm that the following items are present in the figure legend, table legend, main text, or Methods section.

n/a	Confirmed	
☐	☒	The exact sample size (n) for each experimental group/condition, given as a discrete number and unit of measurement
☐	☒	A statement on whether measurements were taken from distinct samples or whether the same sample was measured repeatedly
☐	☒	The statistical test(s) used AND whether they are one- or two-sided<br>*Only common tests should be described solely by name; describe more complex techniques in the Methods section.*
☐	☒	A description of all covariates tested
☐	☒	A description of any assumptions or corrections, such as tests of normality and adjustment for multiple comparisons
☐	☒	A full description of the statistical parameters including central tendency (e.g. means) or other basic estimates (e.g. regression coefficient) AND variation (e.g. standard deviation) or associated estimates of uncertainty (e.g. confidence intervals)
☐	☒	For null hypothesis testing, the test statistic (e.g. F, t, r) with confidence intervals, effect sizes, degrees of freedom and P value noted<br>*Give P values as exact values whenever suitable.*
☒	☐	For Bayesian analysis, information on the choice of priors and Markov chain Monte Carlo settings
☐	☒	For hierarchical and complex designs, identification of the appropriate level for tests and full reporting of outcomes
☒	☐	Estimates of effect sizes (e.g. Cohen's d, Pearson's r), indicating how they were calculated

Our web collection on statistics for biologists contains articles on many of the points above.

Software and code

Policy information about availability of computer code

Data collection	Image acquisition was done using the software NIS-Elements package C.
Data analysis	Image analysis (background subtraction, alignment, and measurements) was performed in Image J 1.52n, and statistical and data analysis was done in R-Studio Version 1.3.1073

For manuscripts utilizing custom algorithms or software that are central to the research but not yet described in published literature, software must be made available to editors and reviewers. We strongly encourage code deposition in a community repository (e.g. GitHub). See the Nature Research guidelines for submitting code & software for further information.

Data

Policy information about availability of data

All manuscripts must include a data availability statement. This statement should provide the following information, where applicable:

- Accession codes, unique identifiers, or web links for publicly available datasets
- A list of figures that have associated raw data
- A description of any restrictions on data availability

• The datasets generated during and/or analysed during the current study are available from the corresponding author on reasonable request

Field-specific reporting

Ecological, evolutionary & environmental sciences study design

All studies must disclose on these points even when the disclosure is negative.

Study description	We tested the effect of pore geometry on the growth of bacteria and fungi and in the bacterial substrate consumption. We used fluorescence microscopy and microfluidics for this purpose. The factors used where Turning angle (45, 90, and 109 degrees), Turning order (alternated, repeated), and competition (presence or absence of the competitor organism). Each microfluidic device contained 10 replicates of each channel type, and 4 microfluidic devices were used per competition treatments (4 for bacteria, 4 for fungi + bacteria, and 4 for fungi).
Research sample	The study was done with the soil bacterial strain Pseudomonas putida, and the litter decomposer fungal strain Coprinopsis cinerea, which were expressing constitutively fluorescent proteins. These species were selected because of their abundance and relevance in soils making them representative of how bacteria and funhi behave in the soil pore space. The population of both organisms increased exponentially in the first days of the experiments, and their growth was measured using fluorescence microscopy.
Sampling strategy	The sample size calculation was done according to the cental-limit theorem (n>30). Our sample size was 720 channels: 40 of each geometrical type (6 types in total) and competition characteristic (3 types).
Data collection	The images of every microfluidic device were taken by the corresponding author with an epi fluorescence microscope every 24 hours.
Timing and spatial scale	The pictures of the experiment were taken every 24 hours from the 17 September 2019, to the 1 of October 2020. The temporal resolution of 24 hours between time point sufficiently allows to appreciate changes in biomass and substrate consumption.
Data exclusions	No data was excluded from the analysis
Reproducibility	The experiment was done previously and the same patterns were found.
Randomization	The channel types were located randomly inside every microfluidic device. Inoculation with bacteria and fungi was done separately to guarantee unbiased inoculation.
Blinding	Blinding was not relevant for the study because the protocol for picture taking and for image analysis is done with the exact same procedure for every image obtained.

Did the study involve field work? ☐ Yes ☒ No

Reporting for specific materials, systems and methods

We require information from authors about some types of materials, experimental systems and methods used in many studies. Here, indicate whether each material, system or method listed is relevant to your study. If you are not sure if a list item applies to your research, read the appropriate section before selecting a response.

Materials & experimental systems

n/a	Involved in the study
☒	☐ Antibodies
☒	☐ Eukaryotic cell lines
☒	☐ Palaeontology and archaeology
☐	☒ Animals and other organisms
☒	☐ Human research participants
☒	☐ Clinical data
☒	☐ Dual use research of concern

Methods

n/a	Involved in the study
☒	☐ ChIP-seq
☒	☐ Flow cytometry
☒	☐ MRI-based neuroimaging

Animals and other organisms

Policy information about studies involving animals; ARRIVE guidelines recommended for reporting animal research

Laboratory animals	No animals were used in these experiments
Wild animals	No wild animals were used in these experiments
Field-collected samples	No field sample collection was done for these experiements
Ethics oversight	No animals were used in these experiments

Note that full information on the approval of the study protocol must also be provided in the manuscript.
