# Supplementary material for: Habitat geometry in artificial microstructure affects bacterial and fungal growth, interactions, and substrate degradation
Source: Commun Biol. 2021 Oct 26;4:1226. doi: 10.1038/s42003-021-02736-4 (PMC8548513; doi:10.1038/s42003-021-02736-4)
Supplement: Supplementary file 1 — Transparent Peer Review File [file 42003_2021_2736_MOESM1_ESM.pdf]

nature portfolio

Peer Review File

Habitat geometry in artificial microstructure affects bacterial and fungal growth, interactions, and substrate degradation

Open Access This file is licensed under a Creative Commons Attribution 4.0 International License, which permits use, sharing, adaptation, distribution and reproduction in any medium or format, as long as you give appropriate credit to the original author(s) and the source, provide a link to the Creative Commons license, and indicate if changes were made. In the cases where the authors are anonymous, such as is the case for the reports of anonymous peer reviewers, author attribution should be to 'Anonymous Referee' followed by a clear attribution to the source work. The images or other third party material in this file are included in the article's Creative Commons license, unless indicated otherwise in a credit line to the material. If material is not included in the article's Creative Commons license and your intended use is not permitted by statutory regulation or exceeds the permitted use, you will need to obtain permission directly from the copyright holder. To view a copy of this license, visit http://creativecommons.org/licenses/by/4.0/.

Reviewers' comments:

Reviewer #1 (Remarks to the Author):

This manuscript developed a microfluidic devices that contained channels differing in bending angles and order to determine the effect of the spatial distortion of a pore space on fungal and bacterial growth, interactions, and substrate degradation. This results can be transferable to soil pore spaces, further to explain organic matter stabilization. I think it would be better if the author explains more on the result section, which can enable readers to understand the effect of the soil pore space.

Other comments:
1, Would you explain why you choose these bacterial (Pseudomonas putida) and fungal (Coprinopsis cinerea)? How about other bacterial and fungal? Is one variety is enough?
2, how to sterilize the microfluidic channels before transferring bacterial? How to minimize possible oxygen contamination when replaced with fresh medium? How often replace during long time (14 days) measurement?

3, Line 145, what kinds of treatment medium?

4, Could you explain "The presence of bacteria caused a reduction of the overall fungal biomass independently of the structures."

5, In figure 3, could you add "a" in the captain of fig. 3a?

6, The author study three angles (45, 90 and 109), but why you plot S. fig. 3 and S. fig. 5 with the angles of 71, 90, and 135?

7, Could you explain why the trend of fluorescent intensity (y axis) of fig. 3 (the bending angle smaller, the intensity higher) is opposite to S. fig. 3, S. fig. 5 and S. fig. 6?

8, Line 266, "increasingly when fungi were present." It is also decreasingly when fungi were present shown on the S. fig.4.

9, Please add the meaning of different color on fluorescence intensity images.

10, what is the pore space in the microfluidic device? Channels?

11, Bacterial accumulation block the pore space? How long has accumulated? Fig. 4 only shows 20 min. Is there possible that the bacterial break through the block if accumulated to a certain amount along with time increased.

Reviewer #2 (Remarks to the Author):

In this article, the authors developed a microfluidic device to determine the effect of the spatial distortion of a pore space on fungal and bacterial growth, interactions, and substrate degradation. Authors have studies that how geometry (by varying angle and order) of microstructures can influence microbial activity. This paper describes a comprehensive study of soil physical parameters of microbial bacteria and fungi. This study is technically sound and would be helpful for scientific readers of soil microbial communities. Thus, I would recommend for its publication with a minor revision. Specific comments are given below

How do authors control the fluids in the microfluidic chip?
How does this chip configuration mimic the actual soil structures and physiology?
As the sharper angles showed reduced bacterial and fungal growth and enzyme activity. How does this chip relevant to actual field conditions full of soil nutrients.
Authors should comment on the effect of soil nutrients in this microfluidic chip.
What are the biotic/abiotic stresses that may occur in this study?

Dear editor and reviewers,

As authors of the present manuscript, we would like to thank you for taking the time to revise our manuscript and, most of all, for providing invaluable comments that will enhance the clearness and scientific value of our work. We have gone through all your comments, concerns, and suggestions, and have included extra information in the manuscript that we hope would suffice for its publication. The text that has been modified is highlighted in yellow in the new version of the manuscript and all changes and responses to each of your comments are addressed in the following table.

Best regards,

Carlos Arellano-Caicedo

Corresponding Author

	Reviewer's comment	Author’s response	Added text in manuscript
**Reviewer 1**	Would you explain why you choose these bacterial (Pseudomonas putida) and fungal (Coprinopsis cinerea)? How about other bacterial and fungal? Is one variety is enough?	These two strains were selected because both are naturally found in soil, thus they can represent, until a certain degree, the behavior of saprotrophic fungi and bacteria. Also, both strains have been widely studied and manipulated, which facilitates the experimental design and the result comparison with other studies. The reasons why the two strains were selected for the experiments are included in the manuscript in lines 166 - 172 and 189 – 192.<br>Of course, including different taxa and a higher number of studied organisms is always better, but the involved experiments are intense and don’t allow screening a large variety of organisms.	**Line 166-172:** " The fungal and the bacterial strain for this experiment were selected based on several criteria, namely the fact that they are naturally soil microorganisms, have both been widely studied, and have thus become model organisms for soil microbes. The bacterial strain used in the experiment was the gram-negative soil bacterium Pseudomonas putida mt-2 carrying plasmid-borne msfGFP-reporter constructs. The reason why this strain was chosen is because its natural habitat is soils, where it is found in high concentrations compared to other strains [38]. Also, its metabolism has been widely studied for bioremediation purposes [39]–[41]. "<br>**Line 189 -192: "** *C. cinerea* is a naturally occurring soil litter decomposer which has been widely studied due to its facility to grow in define medium and be manipulated at any stage of its growth [47]. Also, it has been shown to produce promising results inside microfluidic devices. "

	Reviewer's comment	Author's response	Added text in manuscript
	How to sterilize the microfluidic channels before transferring bacterial? How to minimize possible oxygen contamination when replaced with fresh medium? How often replace during long time (14 days) measurement?	The microfluidic devices were sterilized by exposing them to UV light under a sterile flow bench for 30 minutes. To avoid contamination when filling the devices, all media were previously autoclaved, and the device filling was conducted in a sterile flow bench. Explanation on how the microfluidic devices were sterilized and how the aseptic conditions were kept during the duration of the experiment is included in lines 204-208. The medium was not exchanged during the course of the experiment, oxygen supply was provided through the PDMS and diffusion through the channels. We now explain in more detail in line 161 - 163.	**Line 204-208: "** All the chips and the glass bottomed Petri dishes for experiments with the bacterial and the fungal strain were previously sterilized under UV light in a sterile flow cabinet for 30 minutes. Every step that involved fungal or bacterial inoculation as well as the filling of the chips was done in a sterile flow cabinet. The nutrient medium inside the chips was not replaced after the start of the experiments."<br>.<br>**Line 161-163: "**For the remainder of the experiment, no flow was induced and the liquid medium inside the chip was left to remain static, molecular movement is therefore expected to be dominated by diffusion."
	Line 145, what kinds of treatment medium?	We are using the standard medium M9 amended with the fluorescent substrate as a marker at. The substrate is equal to all treatments, the organism is the treatment factor. A clarification of the used medium is included in line 159-160, and a detailed description of the medium is included in lines 172-176.	**Line 159-160: "** M9 medium with the dissolved fluorogenic reporter was introduced through the reservoir. "
	Could you explain "The presence of bacteria caused a reduction of the overall fungal biomass independently of the structures."	An explanation of this statement is included in lines 303 – 307.	**Line 303-307: "..** meaning that the fungal biomass was equally lower in every channel type when growing with the competitor compared to the corresponding channel when growing alone. This suggests that the effect bacteria had on fungal growth was not due to an interaction with structures that could lead to a habitat modification. "
	In figure 3, could you add "a" in the captain of fig. 3a?	Correction done in figure 3 description.	

	Reviewer's comment	Author's response	Added text in manuscript
	The author study three angles (45, 90 and 109), but why you plot S. fig. 3 and S. fig. 5 with the angles of 71, 90, and 135?	The angle labels were corrected in S Fig 3 and S Fig 5. Thank you for noticing, see also next comment.	
	Could you explain why the trend of fluorescent intensity (y axis) of fig. 3 (the bending angle smaller, the intensity higher) is opposite to S. fig. 3, S. fig. 5 and S. fig. 6?	This error was due to a wrong labelling of the angle axis in fig S3 and S5. The angle labels in both figures were corrected. In the supplementary figures we used angles measured from the opposite side (e.g. 45 vs 135), thus the seemingly opposite results. Thank you for noticing.	
	Line 266, “increasingly when fungi were present.” It is also decreasingly when fungi were present shown on the S. fig.4.	Correction included in line 296-297	**Line 296-297** "… tortuosity in presence and absence of the fungal strain."
	Please add the meaning of different colour on fluorescence intensity images.	Description of each colour was added to Fig. 5 description.	“Figure 5. Examples of fluorescent profiles of *P. putida* expressing GFP constitutively (green), *C. cinerea* expressing d-Tomato constitutively (red), and 4-methylcoumarin (blue) inside the microfluidic channels, on days 2 for *P. putida* and 6 for *C. cinerea* and 4-methylcoumarin, after inoculation inside the microfluidic channels. The fluorescent profiles show the changes in fluorescence along the length of every type of channel studied under the two competitor conditions. The beginning of the channel is located on the left side of each plot.”
	what is the pore space in the microfluidic device? Channels?	The channels are the pore space in the microfluidic device. A clarification is included in the description to Fig. 1.	“Figure 1. Chip design and arrangement of channels. (a) Channel geometries used in the Channel chip. Each channel is characterized by one type of bending angle (45°, 90°, and 109°) and one turn order (alternated or repeated). The channel tortuosity is shown in the last row, which is the ratio between the distance inside the channel between two points and the distance between the same points in a straight line. (b) Chip design, consisting of a pillar system serving as the entry area to the channels, and the array of channels in six variations, randomly distributed along the chip. Structures in

	Reviewer's comment	Author’s response	Added text in manuscript
			black correspond to the open areas of the microfluidic chip pore space in the channels and the pillar system. The chip design dimensions were: 281 mm x 276 mm and the volume of each channel was 2.42 nl. (c) The PDMS chip bonded to a custom-made glass bottom Petri dish, containing the growth medium and microbial cultures introduced via the rectangular reservoir opening.”
	Bacterial accumulation block the pore space? How long has accumulated? Fig. 4 only shows 20 min. Is there possible that the bacterial break through the block if accumulated to a certain amount along with time increased.	Now we mention that during the whole course of the experiment, bacteria were not observed to surpass blockage by fungal hyphae in line 374 - 375. Also, we mention that fungal growth was not observed to be constrained by bacterial accumulations in line 417 - 421.<br>Fig. 4 gives detailed extra information on the processes at cellular scale, and the 20 min are the time it took for the hyphae to grow through the field of view of the camera. The documented growth pattern was observed at many other spots and times and is thus estimated representative.	**Line 374-375: "**Such [fungal] blockages were not observed to be surpassed by bacteria during the duration of the experiment."<br>**Line 417-421:** "When growing with *P. putida*, the effect of structures on fungal growth did not change. This suggests that there was minimal physical effect, such as clogging, caused by bacterial biomass, that could block the advancement of fungal hyphae. Moreover, high magnification videos showed that a bacterial blockage of a channel similar to the one produced by hyphae was not impeding fungal advancement in any of the channels."
**Reviewer 2**	How do authors control the fluids in the microfluidic chip?	The liquid medium was introduced into the microfluidic by pipetting the volume at the entrance of the device. The medium was dragged inside the structures by capillary forces. After filling, the flow inside was not manipulated, meaning that diffusion was the only form of transport of nutrients. A description of how the medium is introduced in the microfluidic device and how the flow regime occurred during the experiment is included in line 159 - 163.	**Line 159-163: "...** M9 medium with the dissolved fluorogenic reporter was introduced through the reservoir. The liquid medium was pipetted into the chip entrance and dragged inside the chip structures by capillary forces. For the remainder of the experiment, no flow was induced and the liquid medium inside the chip was left to remain static, molecular movement is therefore expected to be dominated by diffusion."

	Reviewer's comment	Author’s response	Added text in manuscript
	How does this chip configuration mimic the actual soil structures and physiology?	We chose the approach of using abstract geometrical forms, turning angle and order, for formulation of general predictions of microbial responses to spatial structures that in principle can be found within the broad spectrum of natural spatial structures. An explanation on how the studied structures can mimic specific parameters of soil pore space is mentioned in lines 117 - 122 and 124 - 125.	**Line 117-122: "** The soil pore space in nature has a wide variety of physical parameters, such as the length of its pores, their diameter, and connectivity. In the present study we chose the approach of using abstract geometrical forms for formulation of general predictions of microbial responses to spatial structures, that in principle can be found within the broad spectrum of natural spatial pore space structures. The parameters assigned in this study were the “Angle” and “Turn order” of elongated pore channels."<br>**Line 124-125: "**In this way, we cover the three principal types of angles microorganisms might encounter in the soil pore space: obtuse, right, and acute, respectively."
	As the sharper angles showed reduced bacterial and fungal growth and enzyme activity. How does this chip relevant to actual field conditions full of soil nutrients.	Our results show that sharp angles as well as repeated turn orders of the angles, reduced the bacterial and fungal growth, as well as the nutrients consumed by bacteria inside the channels. According to these results, soils that contain within their pore structures sharp angles with repeated turn orders would have a tendency to have less microbial growth and hence less nutrients consumed. A brief discussion on how the studied parameters would affect microbial communities in a natural soil context is included in line 496 -503	**Line 496-503:** " This would mean, in a natural soil context, that a soil with a more intricate pore space structure including sharper angles would be less successfully explored than in a pore space dominated by less sharp turns, since it would force microbial communities to move or grow backwards to their original direction when exploring the soil pore space. Our results indicate that this is mainly due to the amount of energy for detours that needs to be invested in exploration, which is found to be higher in sharper angles. A reduction in the colonization of a pore space by microbial communities due to sharper angles would thus also lead to a reduction in organic matter turnover in these types of soils. "
	Authors should comment on the effect of soil nutrients in this microfluidic chip.	Soils contain a wider variety of nutrients than the ones we provide in the present experiment. Also, the fluctuations of the different nutrients, as well as their heterogeneous distribution is likely to interact with physical structure, which is something that could be potentially studied in future research. A brief discussion on how soil nutrients might affect the interaction of microbes and their	**Line 527-533:** "Also, the nutrients that microbes encounter in soil are commonly more patchily distributed, have a wider range of chemical complexity and fluctuate more over time than in our experiments where we used a defined medium. This variation in nutrients could interact with structures to enhance or inhibit microbial growth in the pore space. Therefore, experiments testing variation in nutrient proportions that mimic a variety of soil nutrient conditions could focus on studying interaction between nutrient conditions and structures on microbial growth."

	Reviewer's comment	Author’s response	Added text in manuscript
		physical environment is included in line 527 - 533	
	What are the biotic/abiotic stresses that may occur in this study?	The growth conditions of the experiments in terms of nutrients, pH, and temperature were selected to match the optimal growth necessities of the two selected strains, for which reason we believe the only stress that could occur to both strains was due to nutrient depletion, competition, as well as spatial restriction. A brief discussion of possible biotic and abiotic stresses on fungal and bacterial growth in the experiment is included in line 482 - 489	**Line 482-489:** "Finally, *P. putida* and *C. cinerea* seem unlikely to have been affected by additional biotic or abiotic stress since they were growing in a nutrient liquid medium, at room temperature, and a pH that is within the pH range for optimal growth of both strains. The two factors that are affecting both strains growth is either the physical constriction due to the chip structures, and the nutrient depletion that occurs over time. However, other factors that could produce stress, such as accumulation of metabolites or reduction of oxygen levels in the channels, were not measured but could potentially be parameters for future studies."

REVIEWERS' COMMENTS:

Reviewer #1 (Remarks to the Author):

This revised manuscript made an effort to develop a microfluidic device to determine the effect of the spatial distortion of a pore space on fungal and bacterial growth, interactions, and substrate degradation. The author replied to reviewers' comments clearly and modified and highlighted the manuscript. I would suggest accepting for publication in Communications Biology.

Reviewer #2 (Remarks to the Author):

Arellano-Caicedo et al. demonstrated all the questions raised by reviewers in the revised version.

With its current form, I would recommend it for publication.
